# Study of the Metatranscriptome of Eight Social and Solitary Wild Bee Species Reveals Novel Viruses and Bee Parasites

**DOI:** 10.3389/fmicb.2018.00177

**Published:** 2018-02-14

**Authors:** Karel Schoonvaere, Guy Smagghe, Frédéric Francis, Dirk C. de Graaf

**Affiliations:** ^1^Laboratory of Molecular Entomology and Bee Pathology, Department of Biochemistry and Microbiology, Faculty of Sciences, Ghent University, Ghent, Belgium; ^2^Functional and Evolutionary Entomology, Gembloux Agro-Bio Tech, University of Liege, Gembloux, Belgium; ^3^Laboratory of Agrozoology, Department of Crop Protection, Faculty of Bioscience Engineering, Ghent University, Ghent, Belgium

**Keywords:** *Bombus*, *Osmia*, *Andrena*, viruses, bee parasites, metatranscriptomics

## Abstract

Bees are associated with a remarkable diversity of microorganisms, including unicellular parasites, bacteria, fungi, and viruses. The application of next-generation sequencing approaches enables the identification of this rich species composition as well as the discovery of previously unknown associations. Using high-throughput polyadenylated ribonucleic acid (RNA) sequencing, we investigated the metatranscriptome of eight wild bee species (*Andrena cineraria, Andrena fulva, Andrena haemorrhoa, Bombus terrestris, Bombus cryptarum, Bombus pascuorum, Osmia bicornis*, and *Osmia cornuta*) sampled from four different localities in Belgium. Across the RNA sequencing libraries, 88–99% of the taxonomically informative reads were of the host transcriptome. Four viruses with homology to insect pathogens were found including two RNA viruses (belonging to the families *Iflaviridae* and *Tymoviridae* that harbor already viruses of honey bees), a double stranded DNA virus (family *Nudiviridae*) and a single stranded DNA virus (family *Parvoviridae*). In addition, we found genomic sequences of 11 unclassified arthropod viruses (related to negeviruses, sobemoviruses, totiviruses, rhabdoviruses, and mononegaviruses), seven plant pathogenic viruses, and one fungal virus. Interestingly, nege-like viruses appear to be widespread, host-specific, and capable of attaining high copy numbers inside bees. Next to viruses, three novel parasite associations were discovered in wild bees, including *Crithidia pragensis* and a tubulinosematid and a neogregarine parasite. Yeasts of the genus *Metschnikowia* were identified in solitary bees. This study gives a glimpse of the microorganisms and viruses associated with social and solitary wild bees and demonstrates that their diversity exceeds by far the subset of species first discovered in honey bees.

## Introduction

Transcriptomics is the study of all ribonucleic acid (RNA) molecules in a biological sample (Wang et al., 2009). In bees (Hymenoptera; Anthophila clade), transcriptomics has been used to study evolutionary relationships (Peters et al., 2017), eusociality (Woodard et al., 2011), developmental stage and caste differentiation (Harrison et al., 2015), tissue functionality (Yin et al., 2013), and genetic responses to external stimuli (Aufauvre et al., 2014; Riddell et al., 2014). Herein, next-generation sequencing (NGS) followed by bioinformatics analysis of short sequence reads is the most cost-effective method and supplanted previous approaches like expressed sequence tag sequencing or microarrays (Oppenheim et al., 2015). To date, public repositories hold transcriptomics data of at least 62 bee species, the majority of which originate from evolutionary biology initiatives such as the 1K Insect Transcriptome Evolution project (Peters et al., 2017).

Depending on the extraction source, NGS data often comprise a mixture of reads from different species, that is, the host and its co-inhabiting (micro)organisms and viruses (Whitacre et al., 2015). This is notable when the source includes the digestive system (Douglas, 2015) or a specific tissue wherein symbionts reside (DeLay et al., 2012). In case of RNA sequencing, the collection of multi-specific RNA transcripts is then referred to as a metatranscriptome. Where a transcriptomics workflow includes a data preprocessing step to filter out undesired species, such as mapping reads to a database of known co-inhabitants, a metatranscriptomics workflow intentionally retains those reads to simultaneously study species composition and their function. So far, metatranscriptomics studies in bees are limited in number and most are dedicated to the domesticated honey bee, *Apis mellifera*. These studies include investigations of the gut microbiome function (Lee et al., 2015), host-parasite interactions, and viral metagenomics (Runckel et al., 2011; Schoonvaere et al., 2016; Remnant et al., 2017) or both (Cox-Foster et al., 2007; Tozkar et al., 2015).

Bees interact with a remarkable diversity of microorganisms and viruses (Evans and Schwarz, 2011). These interactions constitute either a form of symbiosis with respect to the host (Engel et al., 2016), ranging from beneficial to harmful, or temporarily hitchhiking with respect to a broader plant-pollinator ecology (Lachance, 2016). Bees fall victim to three major groups of unicellular parasites being trypanosomatids (Schmid-Hempel and Tognazzo, 2010), microsporidians (Brown, 2017), and apicomplexans (Lipa and Triggiani, 1996). Also metazoan organisms are common parasites including nematodes (Pionar and Van der Laan, 1972), tracheal mites (Husband and Shina, 1970), and conopid flies (Smith, 1969). Further, honey bees are associated with a variety of viruses, the majority of which bear a single-stranded RNA genome (de Miranda et al., 2013). Despite studies have demonstrated the broad host range of some of those viruses (McMahon et al., 2015; Dolezal et al., 2016) and their capability of inter-taxa replication (Peng et al., 2011), the full impact of bee viruses in a pollinator community is not well understood (Singh et al., 2010).

In this study, we aimed at exploring novel viruses and parasites associated with wild bees using high-throughput RNA sequencing. We adopted a polyadenylation enrichment strategy for two reasons: (1) to selectively enrich for RNA viruses with a polyadenylated genome (which are common in bees) or DNA virus transcripts and (2) to detect as well a significant fraction of the transcriptomes of eukaryote bee parasites and host. We increased the likelihood to encounter infected bees by pooling five individuals per sequencing library and sampling populations from different localities. Despite of the modest selection of only 8 of the nearly 400 wild bee species that occur in Belgium, the diversity of viruses and parasites associated with those species was high and comprised numerous novel associations to bees.

## Materials and Methods

### Sample Collection

**FIGURE 1:**
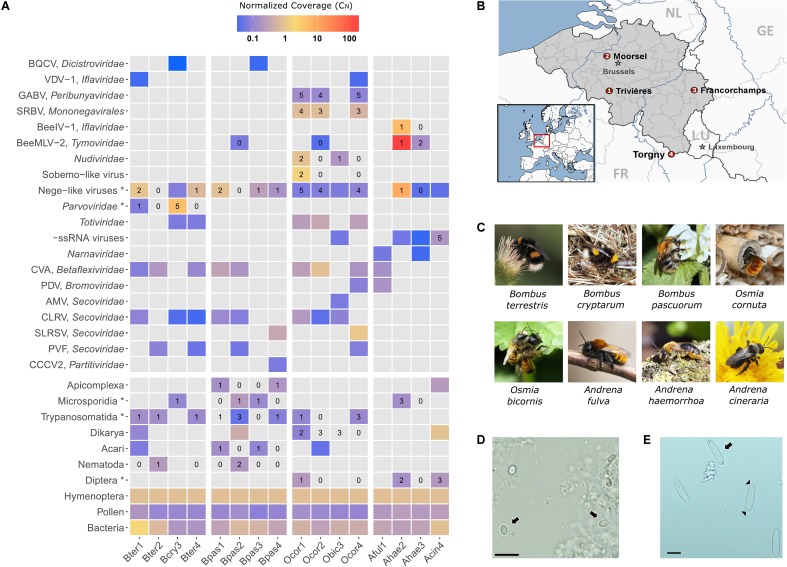
Metagenomics sequencing in eight wild bee species in Belgium. **(A)** Heatmap depicting the relative abundance of the identified bee viruses and other well-supported taxa across the 16 sequencing libraries. Gray-colored tiles indicate that the taxon was not detected by RNA-Seq. Numerical values represent the number of individuals per library (out of five) that were positive by RT-PCR analysis for the corresponding virus or parasite. Taxa marked with an asterisk (^∗^) include more than one species. For example, the taxon “Microsporidia” includes *Nosema bombi, Nosema thomsoni*, and *Tubulinosema* sp. **(B)** Geographical map of Belgium illustrating the four sampling locations (numbers 1–4 correspond to the sequencing libraries). The map was generated using StepMap. **(C)** Natural photographs of the eight different bee species investigated in this study. **(D)** Light microscopic image of spores (arrows) of the parasite *Tubulinosema* sp., bar: 10 μm. **(E)** Light microscopic image of sporocysts (arrows) of the parasite *Apicystis* sp. Note the navicular shape of the sporocysts. Black arrow heads indicate the dense polar plugs at both ends of the sporocyst, bar: 10 μm.

Four sampling locations were selected in correspondence to historical collection sites in Belgium (**Figure 1B**); see also Maebe et al. (2016). At each sampling location, female individuals of the commonest bee species of genera *Bombus, Osmia*, and *Andrena* were collected during the spring of 2015. In case of *Bombus*, only queens that fly early in the bumble bee life cycle could be sampled. Sampling authorization was obtained for protected areas in the southern provinces of the country. We only sampled on days with favorable weather conditions to ensure high bee activity. Bees were individually captured using a sweep net and directly euthanized by placing them on dry ice. At the end of the day, whole bees were stored in the lab at -80°C until further processing. The species determination was confirmed by sequencing of the COI barcode gene from a DNA extract of a single hind leg (see **Supplementary File S1**). Per locality, five individual bees of the same species were selected for RNA extraction. The species included were (**Figure 1C**): *Andrena cineraria* (Acin), *Andrena fulva* (Aful), *Andrena haemorrhoa* (Ahae), *Bombus cryptarum* (Bcry), *Bombus pascuorum* (Bpas), *Bombus terrestris* (Bter), *Osmia bicornis* (Obic), and *Osmia cornuta* (Ocor). The species name abbreviation is extended by a number that refers to one of the four sampling localities, for example, Bter1 refers to *B. terrestris* sampled from locality 1 (**Figure 1B**).

### RNA Extraction and Sequencing

Prior to all tissue handlings, areas and pipettes were wiped with RNase Away^®^ Reagent (Ambion^®^, Life Technologies). We assumed that the head and abdomen tagmata consist of the predominant target tissues for pathogenic viruses and parasites including the alimentary tract, fat bodies, and hypopharyngeal glands. Hence, the thorax tagma was entirely (including wings and legs) left out in order to reduce sample complexity and host fraction. Accordingly, a single bee at a time was dissected on a -80°C surface and tagmata were directly submerged in QIAzol Lysis Reagent (Qiagen). In case of non-corbiculate *Osmia* and *Andrena* bees, external pollen was removed from the abdominal scopa using a sterile knife. Tissues were disrupted using a tissue homogenizer (Precellys) for 90 s at 5000 Hz in the presence of 250 mg 0.1 mm Ø zirconium beads and five 2.3 mm Ø stainless steel beads. RNA was extracted using the RNeasy Lipid Tissue Mini kit (Qiagen) following manufacturer’s instructions including a 15 min on-column DNase I (Qiagen) digestion step. RNA concentration was determined using an ND-1000 UV–Vis Spectrophotometer (Nanodrop Technologies). At this stage, the total RNA was pooled per 5 individuals (10 μg RNA each) per species group and per locality, resulting in 16 pooled total RNA samples. cDNA library construction and sequencing were performed at Omega Bioservices, Norcross, GA, United States (**Supplementary File S1**). The cDNA library preparation method included a polyA+ RNA selective enrichment step and preserved stranded information of the original RNA fragments. An average of 32.7 M (27.2–40.5 M) paired-end 100 bp reads per library was obtained.

### Data Analysis

A detailed report of all *in silico* protocols is given in **Supplementary File S1**. Briefly, after quality assessment, reads were trimmed and *de novo* assembled into contiguous sequences (contigs) using two independent algorithms, CLC Genomics Workbench 9 (Qiagen) and Trinity v2.2.0 (Grabherr et al., 2011). CLC *de novo* contigs were used for homology-based annotation while Trinity contigs were used for validation or refinement purposes. Contigs were queried against the latest versions (August, 2016) of the non-redundant nucleotide and protein sequence databases using BLAST+ v2.2.31 (Camacho et al., 2009) and DIAMOND v0.8.17 (Buchfink et al., 2015), respectively. Blast queries were split over multiple threads using GNU Parallel (Tange, 2011). Annotated contigs were binned based on the lowest common ancestor method implemented in MEGAN6 (Huson et al., 2007) and their fasta sequences extracted for each of the following taxa: Bacteria, Alveolata, Euglenozoa, Dikarya, Microsporidia, Nematoda, Chelicerata, Diptera, Hymenoptera, Viridiplantae, and Viruses. The taxon Viruses was manually refined to the family or species level. Subsequently, nucleotide and protein bins were merged using a custom bash script. Contigs of the taxon Hymenoptera were analyzed by the Benchmarking Universal Single-Copy Orthologs (BUSCO) v2 completeness assessment (Simao et al., 2015). All relevant hits to known or unknown viruses and organisms (except from plants and bacteria) are summarized in **Supplementary Tables S1**, **S2**, respectively. Further, the multi-fasta files per taxon were used as a reference for abundance analysis by aligning forward reads using HISAT2 v2.0.4 (Kim et al., 2015). Count data were normalized to a representative coverage value (scaled by reference length and host average coverage) in R v3.3.0 (R Development Core Team, 2011) and the normalized coverage (*C*_N_) was visualized as a heatmap using ggplot2 v2.2.1 (Wickham, 2009). The *C*_N_ for each taxonomic group provides a rough abundance estimate where a value of 1 represents an equal transcript coverage relative to the host.

### RT-PCR Confirmation and Cloning

All primer sequences and corresponding polymerase chain reaction (PCR) parameters used in this study are included in **Supplementary File S1**. As a general note, PCRs for screening purpose were carried out using HotStartTaq DNA polymerase (DNApol; QIAgen), PCRs for sanger sequencing and GenBank submissions using proof-reading Platinum Pfx DNApol (Thermo Scientific), or in case of difficult templates using expand long template PCR system (Roche). Amplicons were cloned using the TOPO-TA cloning system (Life Technologies) and plasmids were extracted using the GeneJET Plasmid Miniprep Kit (Thermo Scientific). Purified plasmids were sequenced using M13 and custom primers at GATC Biotech (Constance, Germany).

### Virus Genome Organization and Phylogenetic Analyses

Open reading frames (ORFs) were predicted using the online Expasy Translate tool. Conserved domains were predicted from amino acid (aa) sequences using the web-based Interproscan portal. Coverage graphs were exported from CLC Genomics Workbench v8.5 (Qiagen) after mapping paired-end reads to the contigs. Viral taxonomy is based on the International Committee on Taxonomy of Viruses website with the inclusion of the genus type species where possible. All virus abbreviations and accession numbers are summarized in **Supplementary File S2**. The viral replicase protein was used as a phylogenetic marker for RNA viruses, the NS1 protein for ssDNA viruses, and the DNApol, *per os* infectivity factor 2 (pif-2), and late expression factor 8 proteins for dsDNA viruses. Multiple sequence analyses were carried out using MAFFT v7 (Katoh and Standley, 2013) and the L-INS-i (if single domain) or E-INS-i (if multiple domains) iterative refinement methods. The alignment was evaluated (TCS score higher than 5) (Chang et al., 2014) and trimmed. Using the best-fit protein evolution model as determined by ProtTest3 (Darriba et al., 2011), a maximum likelihood tree was estimated using PhyML v3.1 (Guindon et al., 2010) embedded in SeaView (Gouy et al., 2010).

### Data Accessibility

Raw NGS data have been submitted to the SRA repository and can be accessed via the BioProject PRJNA411946. Nucleotide sequences that were cloned and confirmed by Sanger sequencing have been submitted to GenBank under the following name and accessions: *Tubulinosema* sp. isolate Bpas2 (MF998087) and *Apicystis* sp. isolate Bpas1 (MF998086). Other GenBank submissions based on *in silico* assembled contigs are: *A. haemorrhoa* nege-like virus isolate Ahae2 (MF998082), Bee iflavirus 1 isolate Ahae2 (MF998083), Bee macula-like virus 2 isolate Ahae2 (MF998084), and *B. cryptarum* densovirus isolate bcry3 (MF998085). CLC *de novo* and Trinity contigs were stored at a freely accessible Figshare collection (Schoonvaere, 2017), along with taxon-binned multi-fasta sequences, custom bash and R scripts that were generated in this study.

## Results and Discussion

### Host Transcripts Occupied the Highest Proportion of the Metatranscriptome

The host transcriptome fraction, referred to as Hymenoptera, ranged from 88 to 99% of the taxonomically informative reads across the 16 sequencing libraries. To measure the transcriptome completeness of the host, we performed a BUSCO assessment of contigs that were binned to Hymenoptera by MEGAN6 analysis. The different libraries produced a similar profile (**Supplementary Figure S1**) and the average BUSCO notation was C:66.4% [S:66.3%, D:0.1%], F:15.3%, M:18.3%, *n*:4415. In full, out of the 4415 single-copy orthologs in the Hymenoptera lineage, 2930 (66.3%) were complete and single, 5 (0.1%) were complete and duplicated, 667 (15.1%) were fragmented, and 807 (18.3%) were missing. The transcriptome completeness was similar to an independent RNA-Seq study of *B. terrestris* queens (Harrison et al., 2015) indicating that around 70% transcriptome completeness is generally achieved for this caste and sequencing method.

### Insect-Specific Viruses with an RNA Genome

Two honey bee RNA viruses were detected in wild bees. Black queen cell virus (BQCV) was found in sympatric *B. cryptarum* and *B. pascuorum* of a single locality. BQCV was not found elsewhere. In both occurrences, the BQCV read count was very low (**Figure 1A**) and nucleotide (nt) similarity to honey bee BQCV strains was low. Varroa destructor virus-1 (VDV-1) was detected in *B. terrestris* and *O. cornuta*. Read count was very low (**Figure 1A**) but nt similarity to honey bee isolates was high. Intuitively, both BQCV and VDV-1 were present in very low copy number. Either the viruses were at an early infection stage in the bee host or they were associated with pollen (Singh et al., 2010). Two solitary bee viruses that we discovered earlier (Schoonvaere et al., 2016) were also detected here in *O. cornuta*: Scaldis River bee virus (SRBV) and Ganda bee virus (GABV). RT-PCR analysis confirmed SRBV in 66% individuals and GABV in 93% individuals (**Figure 1A**). GABV is related to Apis bunyavirus 2 that was recently discovered in honey bees (Remnant et al., 2017).

**FIGURE 2:**
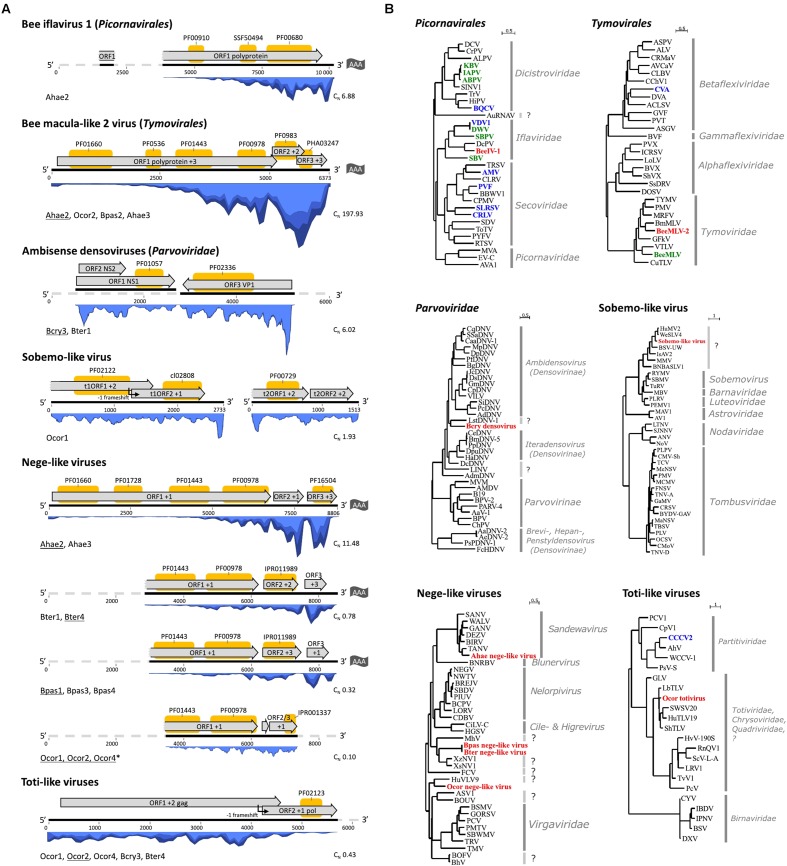
Genome organizations and phylogenetic analyses of wild bee viruses and insect-specific viruses distantly related to plant viruses. **(A)** The genome structure with *in silico* predicted ORF annotations (gray arrow boxes) on the assembled viral transcripts (black lines). In case a genome was not fully recovered by RNA-Seq, the probable complete genome sequence is represented by a gray dashed line. The sequence length in nucleotides is given by the numbers below the lines. Protein domains were predicted using interproscan and are depicted by yellow boxes on top of the ORFs. In blue is the coverage by re-alignment of RNA-Seq reads to the contigs. Below left each map is the sequencing library name wherein the virus was detected and it is underlined in case the depicted transcript originated from that library. ^∗^In case of *O. cornuta* nege-like virus, the contig was obtained by the combined *de novo* assembly of all Ocor datasets. Below right is the normalized coverage corresponding to the heatmap depicted in **Figure 1A**. **(B)** Maximum likelihood trees based on the partial replicase protein (RNA viruses) or non-structural protein 1 (densoviruses). Red abbreviations correspond to the new virus(es) for which a genome organization is given in **(A)**. Blue abbreviations correspond to viruses also detected in the current study but not illustrated in **(A)**. Green abbreviations correspond to known bee pathogenic viruses (not detected in this study). Virus full names and protein accession numbers are included in **Supplementary File S2**.

A novel iflavirus (*Picornavirales*), referred to as bee iflavirus 1 (BeeIV-1), was present in *A. haemorrhoa.* The virus is phylogenetically related to honey bee pathogenic viruses deformed wing virus, sacbrood virus, and slow bee paralysis virus (**Figure 2B**). However, the partial polyprotein of BeeIV-1 had highest aa identity to Dinocampus coccinelae paralysis virus, a virus that induces behavioral changes in its parasitoid host (Dheilly et al., 2015). We should note that the presence of BeeIV-1 in *A. haemorrhoa* was confounded by the co-infection of a conopid fly larva and a microsporidian. The possibility exists that BeeIV-1 was present as an infection of the fly larva rather than the bee host. However, BeeIV-1 was absent from another conopid-infected *A. haemorrhoa* individual suggesting no obligate relationship between BeeIV-1 and parasitic flies.

A novel macula-like virus (*Tymovirales*), referred to as bee macula-like virus 2 (BeeMLV-2), was detected in multiple hosts *B. pascuorum, O. cornuta*, and *A. haemorrhoa* but was only present in high copy number in library Ahae2 (**Figure 1A**). The recovered genome sequence of BeeMLV-2 was nearly complete and a similar organization was observed compared to related macula-like viruses (**Figure 2A**). Based on RdRp protein phylogeny, BeeMLV-2 clustered close to members of *Maculavirus* in the family *Tymoviridae* and its closest relative was Bombyx mori macula-like virus (**Figure 2B**). This virus was originally identified in cell cultures (Katsuma et al., 2005). BeeMLV-2 was more distantly related to bee macula-like virus (BeeMLV) that is currently unassigned in *Tymoviridae* awaiting genus designation. BeeMLV is a pathogen of honey bees and the parasitic mite *Varroa destructor* (de Miranda et al., 2015).

Two contigs in *O. cornuta* library Ocor2 showed homology to arthropod-infecting +ssRNA viruses that are distantly related to sobemoviruses. Although the primary hosts of sobemoviruses are plants, sobemo-like viruses have been reported in fruit flies (Webster et al., 2016), mosquitoes (Shi et al., 2016), ants, and mites (Tokarz et al., 2014). Phylogenetic analysis based on the replicase protein placed the sobemo-like virus together with other arthropod viruses in a clade close to *Luteoviridae* (**Figure 2B**). The longest transcript had a similar genome organization to that of Ixodes scapularis associated virus-2 where the viral serine protease ORF1 overlaps the RdRp protein ORF2, a feature that is shared among sobemoviruses.

Contigs related to insect-specific negeviruses and plant viruses of the family *Virgaviridae* were identified in all libraries except Aful1, Bter2, and Bpas2. Each bee species was associated with a particular nege-like virus. The genome sequence of viruses from different localities shared less than 1% sequence dissimilarity, which can be attributed to the natural variation in RNA viruses. The genome organization of four nege-like viruses are depicted in **Figure 2A**. Comparison of nege-like viruses from different bee species revealed strong analogy in genome organization and evolutionary relationship (**Figure 2B**). Based on RdRp protein phylogeny, the *A. haemorrhoa* nege-like virus was related to the Sandewavirus phylogenetic group of negeviruses. The RdRp protein was most similar (49% overall aa identity) to Tanay virus, a nege-like virus of mosquitoes (Nabeshima et al., 2014). The *O. cornuta* nege-like virus was phylogenetically related to nege-like viruses of the moth *Epirrita autumnata* (de Miranda et al., 2017), dipterans (Shi et al., 2016; Webster et al., 2016), and the plant bug *Adelphocoris suturalis* (Li et al., 2017). *Bombus terrestris* and *B. pascuorum* nege-like viruses were most related to viruses associated with nematodes (Shi et al., 2016). The viruses in *B. terrestris* and *B. pascuorum* were highly similar to each other (ORF1-3 predicted viral proteins had 92, 86, and 91% protein identity, respectively). It is worth noting that the nege-viral sequences were not detected in nematode-associated *B. terrestris* and *B. pascuorum* libraries (locality 2), despite of their relatively abundant presence in nematode-free libraries.

In all *O. cornuta* libraries, one or two sets of contigs had homology to totiviruses. The most representative genome sequence was obtained in library Ocor2 with a length of 6.6 Kb (**Figure 2A**). Genome organization is similar to that of Leptopilina boulardi toti-like virus (LbTV) where ORF1 overlaps ORF2 and translation of ORF2 is mediated by a -1 ribosomal frameshift. Sequences of libraries Ocor1 and Ocor2 are very similar (99% nt identity), but both are more dissimilar to the totivirus in library Ocor4 (93% nt identity) suggesting that different strains exist. Members of the family *Totiviridae* have a linear dsRNA genome of 4.6–6.7 Kb in size and are originally described from fungal hosts. However, several insect totiviruses have been discovered that currently fall apart in two groups, the one infecting Hymenoptera including parasitic wasps (Martinez et al., 2016) and ants (Koyama et al., 2015) and the other infecting Diptera including mosquitoes (Zhai et al., 2010) and fruit flies (Wu et al., 2010). The RdRp and capsid proteins predicted from the toti-like viral sequences in Ocor2 showed highest homology to a member of the first group (**Figure 2B**). In fact, Martinez et al. (Martinez et al., 2016) already showed the presence of fragmentary transcripts in *O. cornuta* transcriptome shotgun assembly data related to LbTV. Experimental studies with LbTV in a parasitoid wasp system showed that it is biparentally transmitted (through both eggs and sperm) and that infection positively correlates with developmental success (Martinez et al., 2016).

Sequences of –ssRNA viruses were too fragmented in order to discuss their genome organization and phylogeny. Two contigs of *A. haemorrhoa* had 68–70% aa identity to viruses of the genus *Quaranjavirus*, family *Orthomyxoviridae*. Eleven contigs (summed length 8837 nt) of *O. bicornis* consistently had around 80% aa identity to SRBV RdRp, nucleoprotein, and glycoprotein. Nine contigs (summed length 6394 nt) of *A. cineraria* had 36–42% aa identity to RdRp proteins of rhabdoviruses (*Mononegavirales*) including Wuhan ant virus and Apis rhabdovirus 1 and 2. Both rhabdoviruses were shown to elicit an anti-viral immune response in honey bees (Remnant et al., 2017). The rhabdo-like virus was confirmed to be present in all five *A. cineraria* individuals. Together, these reports add to the growing evidence that –ssRNA viruses are widespread in arthropods (Li et al., 2015) including bees (Schoonvaere et al., 2016; Remnant et al., 2017).

### Insect-Specific Viruses with a DNA Genome

**FIGURE 3:**
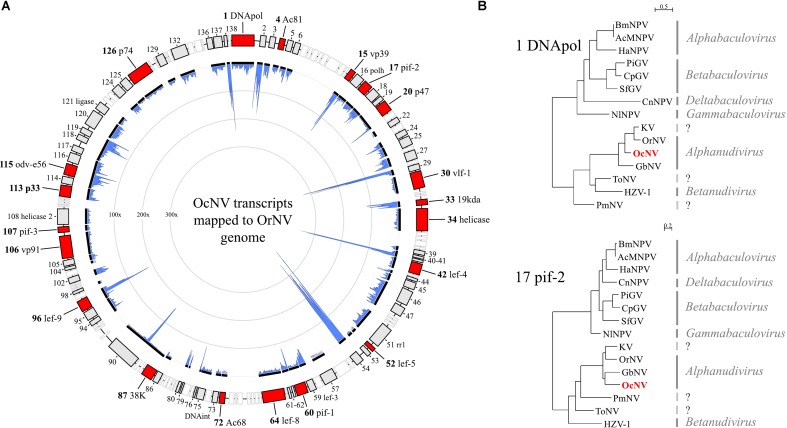
The transcriptome and phylogenetic analysis of *Osmia cornuta* nudivirus (OcNV). **(A)** Nudiviral transcripts (black lines) were mapped to the genome map of *Oryctes rhinoceros* nudivirus (OrNV). Note that the order of the transcripts is not representative for OcNV since genomic reorganizations are common in dsDNA viruses. In total, 74 orthologs with respect to OrNV genes (boxes) were identified. Red boxes represent the 20 core baculoviral genes that are shared among *Baculoviridae* and *Nudiviridae*. Empty (white) boxes represent OcNV genes that were not found in the OrNV transcriptome. In blue is the coverage (100–300x) by realignment of RNA-Seq reads to the contigs which correlates to the abundance of the viral transcript in the library Ocor2. **(B)** Maximum likelihood trees of OcNV based on DNApol and pif-2 conserved domains pfam03104 and pfam04631, respectively. The phylogenetic analyses included viruses of the families *Baculoviridae* (four genera) and *Nudiviridae* (two genera). A third analysis with late expression factor 8 protein gave a tree with a similar topology to that of DNApol and was considered redundant. Both trees depicted here placed OcNV close to OrNV and *Gryllus bimaculatus* nudivirus of the genus *Alphanudivirus*. Virus full names and protein accession numbers are included in **Supplementary File S2**.

A dsDNA virus belonging to the *Nudiviridae* family was present in allopatric *O. cornuta* and *O. bicornis* females. The same virus was encountered in a previous metagenomic survey (Schoonvaere et al., 2016). We confirmed that both reports concern the same virus, which we tentatively name *O. cornuta* nudivirus (OcNV). OcNV is phylogenetically related to pathogenic nudiviruses in beetles (Huger, 2005), crickets (Huger, 1985), and dipterans (Webster et al., 2015) that belong to the genus *Alphanudivirus* (**Figure 3B**). Because it is not possible to obtain the complete genome sequence of a DNA virus by RNA sequencing, we mapped the OcNV transcriptome to the complete genome of a related nudivirus (**Figure 3A**) to estimate its completeness. Strikingly, OcNV homologs to all of the 20 core baculoviral genes shared among nudiviruses and baculoviruses (Wang et al., 2012) were expressed. The summed transcript length of the 74 nudivirus orthologs identified in library Ocor1 was 94.4 Kbp which is nearly the genome length of Kallithea virus (96.9 Kbp). We expect that the genome of OcNV exceeds 100 Kbp because there are likely more OcNV-specific proteins that were not detected here. The abundant presence of viral RNA transcripts that originate from a common DNA template at least indicates that OcNV is actively replicating inside bees. Further, the relatively low number of OcNV positive bees in spatially separated populations of two closely related bee species suggests a specialized relationship, though further studies need to confirm this hypothesis. Since *O. cornuta* and *O. bicornis* are commercially distributed pollinators in Europe, caution should be taken in the trade of bees that carry OcNV before more is known about possible pathogenicity and reproductive success effects of this virus.

*Bombus cryptarum* (Bcry3) and one *B. terrestris* library (Bter1) were associated with viral sequences of ssDNA viruses of the family *Parvoviridae*. The closest relative (36% protein identity) was the ambidensovirus Diaphorina citri densovirus (DcDNV), although phylogenetic analysis of the replicase protein placed the *B. cryptarum* densovirus together with Lone star tick densovirus 1 (LstDNV-1) in a separate clade (**Figure 2B**). A characteristic feature of ambidensoviruses is that structural (VP) and non-structural (NS) cassettes are transcribed from opposite DNA strands. In case of DcDNV, the VP and NS cassettes are separated by 21 nt (Nigg et al., 2016). Since we initially identified full-length VP and NS densoviral ORFs in two Bcry3 transcripts that could not be linked by paired-end RNA-Seq read data (**Figure 2A**), we anticipated a non-overlapping, ambisense genome organization similar to that of DcDNV. Indeed, gap-spanning PCR on a genomic DNA extract of an infected bee and Sanger sequencing of the amplicon confirmed that both transcripts originate from the same DNA template but are in opposite orientation and separated by 30 nt (the actual number depends on the true size of the viral transcripts). Densoviral contigs were also found in library Bter1 with 70–75 and 88% nucleotide identity to the densoviral NS and VP transcripts in Bcry3, respectively, although the coverage was much lower. RT-PCR revealed that all (5) *B. cryptarum* queens and one *B. terrestris* queen were associated with a densovirus (**Figure 1A**). Despite of the broad host range of densoviruses in insects, none so far have been reported in bees. The relation of densoviruses of the subfamily *Densovirinae* to their arthropod host ranges from mutualistic (Xu et al., 2014) to severe pathology (Dhar et al., 2014), which is especially problematic in large insect rearing facilities (Szelei et al., 2011). In this respect, it is worth to investigate the biological relationship between the densoviruses that were discovered here and the commercial pollinator species *B. terrestris*.

### Plant and Fungal Viruses

Seven well-characterized plant viruses were detected: cherry virus A (CVA), Prunus dwarf virus (PDV), cherry leaf roll virus (CLRV), Prunus virus F (PVF), strawberry latent ringspot virus (SLRSV), arabis mosaic virus (AMV), and crimson clover cryptic virus 2 (CCCV-2). Except from CCCV-2, all are +ssRNA viruses and some are serious pathogens of crops (Cooper, 1979). Only the genome of PDV is not polyadenylated. The normalized coverage of the plant viruses was low to moderate in comparison to most insect-specific viruses; however, CVA and SLRSV were, respectively, more abundant in Ocor2 and Ocor4 libraries (**Figure 1A**). Because the primary source of plant viruses in bees is likely to be dietary pollen, we anticipated a correlation between the abundance of plant virus and pollen-derived reads. Surprisingly, no plant viruses were detected in *A. haemorrhoa* and *A. cineraria* despite of the larger proportion of pollen-derived reads in these hosts. Four of the plant viruses detected in this study belong to the family *Secoviridae* (order *Picornavirales*) of which AMV and CLRV are nepoviruses. Recently, a plant pathogenic nepovirus (*Secoviridae*) was demonstrated to replicate in and negatively affect honey bees (Li et al., 2014). We could not infer from our data whether these viruses replicated in bees. However, CLRV was detected in each locality and associated with all species except *Andrena* spp.

In two *Andrena* libraries, low-abundant sequences were detected with up to 40% protein homology to narna-like viruses isolated from yeasts and crustaceans. Narnaviruses (*Narnaviridae*) have a monopartite, linear +ssRNA genome of 2.5–2.9 Kb in size and naturally infect fungi. Two fragmentary, narna-like viral contigs in *A. fulva* had a summed length of 2077 nt and a candidate partial genome sequence of 2.6 Kb was manually refined.

### Typical Bee Parasites: Trypanosomes, Microsporidia, and Neogregarines

The trypanosome *Crithidia bombi* was commonly detected in *B. terrestris*, *B. pascuorum*, and *O. cornuta* (**Figure 1A**), and typically occupied less than 0.6% of sequencing libraries. A trypanosome nearly identical to *Crithidia pragensis* was identified in *O. cornuta* (**Table 1**). *Crithidia pragensis* is originally described from the fly *Cordilura albipes* in Central Europe (Yurchenko et al., 2014). The library fraction that was occupied by *C. pragensis* was similar to that of *C. bombi* indicating that it was present as an internal parasite. There was no evidence for a co-infection of a parasitic fly species although the same individual carried the viruses OcNV, GABV, SRBV, *O. cornuta* nege-like virus, and a sobemo-like virus. The microsporidian *Nosema bombi* was found in sympatric *B. cryptarum* and *B. pascuorum* but not elsewhere (**Figure 1A**). Either the microsporidium *Nosema thomsoni* or a very related species was associated with *A. haemorrhoa* (**Figure 1A**). It was most identical to the *Nosema* associated with *Andrena vaga* (Ravoet et al., 2014). A third microsporidian transcriptome was detected in library Bpas2 that was clearly different from *Nosema*. The partial ribosomal DNA cassette of the microsporidium was cloned and its SSU rRNA sequence was equally similar to that of *Tubulinosema pampeana* and *Tubulinosema loxostegi* (**Table 2**), which, respectively, are low-virulent parasites recently described from *Bombus atratus* in S-America (Plischuk et al., 2015) and the moth *Loxostege sticticalis* in W-Siberia (Malysh et al., 2013). The *Tubulinosema* infected bumble bee was co-infected with *C. bombi* and the nematode *Sphaerularia bombi*. Ovoid morphologies that strongly resemble the spores of *T. pampeana* were observed in the thorax of the infected bumble bee (**Figure 1D**). An apicomplexan parasite equally similar to *Apicystis bombi* as *Mattesia geminata* (**Table 3**) was associated with two *B. pascuorum* libraries. However, microscopical examination of the infected thoraces revealed morphisms similar in shape as the navicular sporocysts of *A. bombi* (**Figure 1E**).

**Table 1:** Sequence similarity matrix of *C. pragensis* and related trypanosomes.

Species	Host	1	2	3	4	5	6	7	8	9	10	11
(1) *Crithidia* sp.	Hymenoptera: Megachilidae		99,4	99,0	99,0	96,8	96,1	97,4	95,5	94,2	94,9	96,8
(2) *C. pragensis* MCZ-11	Diptera: Scathophagidae	99,8		98,4	98,4	96,1	95,5	96,8	94,9	93,6	94,2	96,1
(3) *Leptomonas* sp. Cfm	Hemiptera: Nabidae	98,8	98,6		98,7	96,8	96,1	97,4	95,5	94,2	94,9	96,8
(4) *C. permixta* 128SI	Hemiptera: Miridae	97,4	97,2	96,5		96,5	95,8	97,1	95,2	93,9	94,5	96,5
(5) *L. tarcoles*	Hemiptera: Miridae	94,3	94,1	93,9	93,3		97,1	97,4	95,8	95,2	94,9	96,5
(6) *C. brevicula* ZK c2	Hemiptera: Nabidae	94,0	93,8	93,6	93,2	94,7		96,8	94,2	98,1	93,6	95,5
(7) *C. fasciculata*	Diptera: Culicidae	94,0	93,8	93,7	92,9	97,5	94,3		96,8	94,9	94,5	96,8
(8) *C. mellificae* 277-1+2h	Hymenoptera: Apidae	93,7	93,5	92,9	93,0	93,4	93,3	93,5		92,3	95,5	97,1
(9) *Blastocrithidia gerricola*	Hemiptera: Gerridae	93,3	93,1	92,8	92,4	94,1	99,3	93,8	92,7		91,6	93,6
(10) *Lotmaria passim* 277-3+4c(x2)	Hymenoptera: Apidae	91,9	91,7	91,2	91,0	93,0	91,7	93,6	93,2	91,1		96,5
(11) *C. bombi* Ocor4	Hymenoptera: Megachilidae	92,1	91,9	91,3	91,7	92,1	91,9	92,5	94,3	91,1	93,9	


*Similarity percentages were calculated for the glycosomal glyceraldehyde dehydrogenase nucleotide (935 nt, lower) and protein (311 aa, upper) sequence using MatGAT v2.0.1. The trypanosome in *O. cornuta* is most related to *C. pragensis*.*

**Table 2:** Sequence similarity matrix of *Tubulinosema* sp. and related apicomplexans.

Species	Host	1	2	3	4	5	6	7	8	9	10	11
(1) *Tubulinosema* sp.	Hymenoptera: Apidae											
(2) *T. pampeana*	Hymenoptera: Apidae	97,9										
(3) *T. loxostegi*	Lepidoptera: Crambidae	97,9	96,8									
(4) *T. hippodamiae*	Coleoptera: Coccinellidae	97,3	97,1	97,6								
(5) *T. ratisbonensis*	Diptera: Drosophilidae	96,8	95,9	97,7	97,6							
(6) *T. kingi*	Diptera: Drosophilidae	95,6	94,8	96,6	96,4	98,5						
(7) *Fibrillanosema crangonycis*	Amphipoda: Crangonyctidae	78,2	77,6	78,2	77,3	77,9	77,3					
(8) *Kneallhazia solenopsae*	Hymenoptera: Formicidae	74,3	74,4	74,3	73,9	74,0	73,4	73,8				
(9) *Bryonosema plumatellae*	Bryozoa: Plumatellidae	71,6	71,3	71,1	71,4	71,3	70,8	71,7	67,6			
(10) *Anncaliia algerae*	Insects (mosquitoes), human	72,3	72,4	72,3	72,7	72,6	72,5	73,5	81,0	66,5		
(11) *A. meligethi*	Coleoptera: Nitidulidae	69,7	69,1	69,9	69,4	69,6	69,6	70,5	78,0	64,1	91,9	
(12) *Pseudonosema cristatellae*	Bryozoa: Cristatellidae	70,2	70,3	69,8	70,3	70,0	70,3	69,8	66,6	79,9	65,9	63,6


*Similarity percentages were computed for a partial region of the small subunit RNA gene of Microsporidia spp. using MatGAT v2.0.1. The region corresponds to KM998087(828..1823). The novel tubulinosematid in *B. pascuorum* is equally similar to *T. pampeana* and *T. loxostegi*.*

**Table 3:** Sequence similarity matrix of *Apicystis* sp. and related neogregarines.

Species	Host	1	2	3	4	5	6	7	8	9	10	11
(1) *Apicystis* sp.	Hymenoptera: Apidae											
(2) *Apicystis bombi*	Hymenoptera: Apidae	95,6										
(3) *Mattesia geminata*	Hymenoptera: Formicidae	95,6	94,2									
(4) *M.* sp. SV-2003	Hymenoptera: Formicidae	95,2	94,1	98,2								
(5) *Ascogregarina culicis*	Diptera: Culicidae	88,4	88,4	89,1	88,8							
(6) *As. taiwanensis*	Diptera: Culicidae	88,3	88,6	89,3	88,9	99,4						
(7) Apicomplexan sp.	Astigmata: Acaridae	88,3	87,7	88,1	88,3	94,2	94,6					
(8) *Ophriocystis elektroscirrha*	Lepidoptera: Nymphalidae	87,9	88,7	88,1	88,0	91,4	91,4	92,2				
(9) *Aranciocystis muskarensis*	Coleoptera: Scarabaeidae	87,9	88,1	88,6	88,5	93,6	93,8	97,4	91,8			
(10) *Paraschneideria metamorphosa*	Diptera: Sciaridae	87,7	88,4	88,6	88,2	95,0	95,1	93,5	90,8	92,8		
(11) *Syncystis mirabilis*	Hemiptera: Nepidae	86,6	86,6	87,5	87,0	89,6	89,6	88,6	86,8	88,6	89,8	
(12) *Geneiorhynchus manifestus*	Odonata: Aeshnidae	85,9	85,8	86,9	86,2	88,2	88,3	87,3	87,2	87,6	88,0	87,2


*Similarity percentages were computed for a partial region (1170 nt) of the small subunit RNA gene of Gregarinasina spp. using MatGAT v2.0.1. The region corresponds to KM998086(469..1638). The novel neogregarine of *B. pascuorum* is equally similar to *A. bombi* and *M. geminate*.*

### Ascomycetous Yeasts and Basidiomycete Fungi

The summit of an ascomycetous yeast transcriptome was observed. About 100 protein-coding transcripts were annotated. The four most abundant transcripts were cytochrome C oxidase subunits I and II, elongation factor 1 alpha, and a mitochondrial carrier protein. Based on sequence similarity of the 5′ partial large subunit RNA and small subunit RNA, the closest related species were *Metschnikowia pulcherima*, *Metschnikowia chrysoperlae*, and *Metschnikowia* (*Candida*) *pimensis*. All are members of the *M. pulcherima* clade which is primarily associated with nectar and fruit-feeding insects (Lachance, 2016). Some members of this clade produce pulcherrimin, an iron-chelating molecule that induces antagonistic activity against fungi and bacteria (Sipiczki, 2006). Other clades are pathogens of marine arthropods or are strictly associated with beetles (Lachance, 2016). Yeasts of the genus *Metschnikowia* are not new to bees. In fact, it was long assumed that a mutualistic relation exists between both, wherein the yeast hibernates inside its bee host, although recent experimental evidence contradicts this hypothesis (Brysch-Herzberg, 2004). The size of the yeast transcriptome detected here is similar to that of internal bee parasites such as *Crithidia* and *Nosema* spp. and at least indicates that the yeasts cells were abundantly present. *Metschnikowia* contigs were also found in library Acin4 but only originated from ribosomal RNA genes. Further, several contigs were found in libraries Bter1 and Bpas2 that had annotations to basidiomycete genes including *Hypsizygus marmoreus*, *Serpula himantioides*, *Pycnoporus cocci*, and *Pleurotus* sp. The significance of these associations is likely very low and probably results from external contaminants.

### Metazoan Bee Parasites

The parasitic nematode *S. bombi* was present in one *B. terrestris* queen and two *B. pascuorum* queens of a single locality (Bter2 and Bpas2), but was absent elsewhere (**Figure 1A**). The nematode transcriptome fraction occupied 5.5 and 2.3% of the sequencing library, respectively. Sequence comparison of conserved house-keeping transcripts revealed that *S. bombi* of interspecific hosts was genetically distinct, which suggests a host-specific haplotype structuring of this parasite. Further, the tracheal mite *Locustacarus buchneri* was molecularly detected in *B. pascuorum* queens (**Figure 1A**). Finally, the larvae of two conopid fly species, *Myopa testacea* and *Myopa tesselatipennis*, were detected as abdominal parasites. *Myopa testacea* was present in one *O. cornuta* female and three *A. cineraria* females whereas *M. tesselatipennis* was present in two *A. haemorrhoa* females (**Figure 1A**). The fly transcriptome typically occupied less than 3% of the sequencing library.

## Conclusion

In this study, we combined a polyadenylated RNA enrichment with high-throughput RNA sequencing to explore the metatranscriptome of eight social and solitary wild bee species, of which the transcriptomes of five species (*A. cineraria, A. haemorrhoa, A. fulva, B. cryptarum*, and *B. pascuorum*) were not sequenced earlier. We identified over 20 associated viruses, 10 different species of bee parasites, and associated fungi. Two unknown RNA viruses in *A. haemorrhoa* (bee iflavirus 1 and bee macula-like virus 2) and two unknown DNA viruses in *Osmia* spp. and *Bombus* spp. (OcNV and a densovirus, respectively) were related to other pathogenic viruses of insects. These viruses may be of pathological importance to the studied bees and other bee species, or in case of the DNA viruses to the rearing of the commercial pollinator species *O. cornuta*, *O. bicornis*, and *B. terrestris*. However, the potential pathological association of the viruses has to be validated by follow-up studies that include isolation of the virus particles and experimental inoculation in the bee host. We also discovered the widespread occurrence of nege-like viruses, negative-stranded RNA viruses, and other insect-specific viruses, which is in congruence with recent large-scale, viral metagenomics studies in arthropods. However, the role of these viruses and their significance with respect to insects in general is unknown. Further, next to typical bee parasites, we detected for the first time in Europe a *Tubulinosema* parasite in a bumble bee host, a fly trypanosome parasite that infected *O. cornuta*, and a neogregarine parasite that is related but molecularly dissimilar to *A. bombi*. We encourage future prevalence studies to include the novel associations of viruses and parasites reported here to learn more about their geographical distribution and impact on pollinator health.

## Author Contributions

DdG, GS, and KS conceived the study. KS performed the sampling and practical work. DdG, FF, and KS wrote the manuscript. DdG, GS, FF, and KS revised the manuscript.

## Conflict of Interest Statement

The authors declare that the research was conducted in the absence of any commercial or financial relationships that could be construed as a potential conflict of interest.

## Supplementary Material

**FIGURE S1 |** BUSCO completeness assessment.

**TABLE S1 |** Description of viruses associated with wild bees.

**TABLE S2 |** Description of protozoan and metazoan parasites associated with wild bees.

**FILE S1 |** Supplementary information for Materials and Methods.

**FILE S2 |** Virus abbreviations and protein accessions used in phylogenetics analyses.
