# Supplementary material for: Study of the Metatranscriptome of Eight Social and Solitary Wild Bee Species Reveals Novel Viruses and Bee Parasites
Source: Front Microbiol. 2018 Feb 14;9:177. doi: 10.3389/fmicb.2018.00177 (PMC5817871; doi:10.3389/fmicb.2018.00177)

Figure S1. BUSCO Completeness assessment.

An average transcriptome completeness (light blue) of 66.4 % was obtained across RNA-Seq libraries. This was comparable to a different RNA-Seq study (Harrison et al., 2015) in bumble bee queens of which we downloaded the raw data from the Sequence Read Archive (SRA) and inputted to our metagenomics pipeline. BUSCO analysis was also run on a consortium dataset of Bombus terrestris v1.3 predicted transcripts that we downloaded from BEEBASE (hymenopteragenome.org/beebase) and resulted in a 94.1 % completeness.

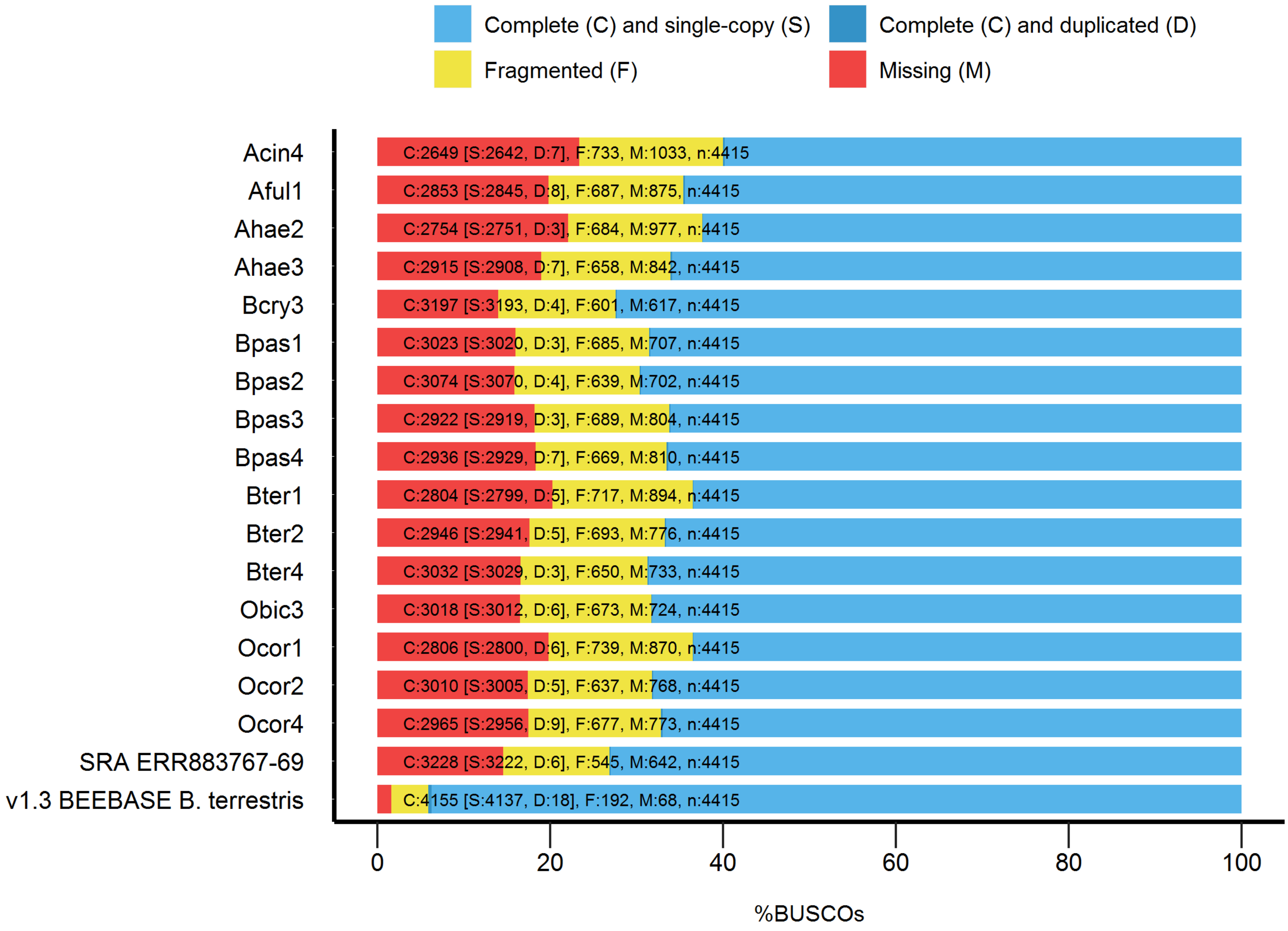

Supplement: Supplementary file 1 [file Image_1.PDF]
