# Supplementary material for: Study of the Metatranscriptome of Eight Social and Solitary Wild Bee Species Reveals Novel Viruses and Bee Parasites
Source: Front Microbiol. 2018 Feb 14;9:177. doi: 10.3389/fmicb.2018.00177 (PMC5817871; doi:10.3389/fmicb.2018.00177)
Supplement: Supplementary file 2 [file Table_1.DOCX]

Table S1. Description of viruses associated with wild bees.

CLC/Trinity contigs are available from <https://figshare.com/s/13f0bac389b1188133d5>

| Name | Current Taxonomy | RNA-Seq + | RT-PCR + | Description |
| --- | --- | --- | --- | --- |
| Black queen cell virus (BQCV) | +ssRNA virus  Picornavirales  *Dicistroviridae*  *Triatovirus* | Bcry3  Bpas3 | ns | A honey bee (*Apis mellifera*) pathogenic virus causing mortality in queen and worker pupae (Bailey & Woods, 1977). Multiple BQCV strains known from all over the world. Able to infect *Bombus huntii* of N-America (Peng et al., 2011). Detected (+ve strand) in *Osmia cornuta*, *Andrena vaga* and *Heriades truncorum* in Europe (Ravoet et al., 2014). Singh and co-workers (Singh et al., 2010) detected (+ve strand) BQCV in *Bombus* sp., *Andrena* sp. and *Xylocopa virginica* in N-America and simultaneously showed that BQCV is strongly associated with pollen. |
| Varroa destructor virus 1 (VDV-1) | +ssRNA virus  Picornavirales  *Iflaviridae*  *Iflavirus* | Bter1  Ocor4 | ns | A virus of the honey bee pathogenic mite *Varroa destructor* and highly related to honey bee viruses Deformed wing virus and Kakugo virus (Ongus et al., 2004). Together “DWV-complex” viruses. Later, VDV-1 was shown also to replicate in the head of honey bees (Zioni, Soroker, & Chejanovsky, 2011). Recently found in *O. cornuta* by current authors (Schoonvaere et al., 2016). |
| Ganda bee virus (GABV) | -ssRNA virus  Bunyavirales  *Peribunyaviridae*  unclassified | Ocor1  Ocor2  Ocor4 | 4, 13, 14, 100, 101, 28, 33, 35, 37, 74, 77, 78, 79, 81 | Novel bee virus detected by metagenomics survey in *O. cornuta* (Schoonvaere et al., 2016) (KY053854-56), demonstration of active replication in the bee host. Biological role unknown. The virus has not yet been isolated nor shown to be pathogenic to the bee. Detected by RT-PCR in head, thorax, abdomen and eggs of *O. cornuta* females (unpublished results), suggesting vertical transmission of this virus. |
| Scaldis River bee virus (SRBV) | -ssRNA virus  Mononegavirales  unassigned | Ocor1  Ocor2  Ocor4 | 4, 13, 14, 100, 28, 34, 35, 77, 78, 79 | Novel bee virus detected by metagenomics survey in *O. cornuta* (Schoonvaere et al., 2016) (KY053857), demonstration of active replication in the bee host. Biological role unknown. Detected by RT-PCR in head, thorax, abdomen and eggs of *O. cornuta females* as well as in pollen provisions (unpublished results). |
| Bee iflavirus 1 (BeeIV-1) | +ssRNA virus  Picornavirales  *Iflaviridae*  *Iflavirus* | Ahae2 | 157 | Iflavirus closely related to Wuhan insect virus 13 (NC_033455) (Shi et al., 2016) and *Dinocampus coccinellae* paralysis virus (NC_025835) (Dheilly et al., 2015). More distantly related to DWV-complex viruses, Slow bee paralysis virus and Sacbrood virus. Here, BeeIV-1 was co-infected with two other viruses, BeeMLV-2 and *Andrena haemorrhoa* nege-like virus. |
| Bee macula-like virus 2 (BeeMLV-2) | +ssRNA virus  Tymovirales  *Tymoviridae*  unclassified | Bpas2  Ocor2  Ahae2 Ahae3 | 157, 126, 130 | Putative maculavirus most similar to *Bombyx mori* macula-like virus (BmMLV, NC_015524) isolated from silk moth cell lines (Katsuma et al., 2005). Phylogenetically unresolved but most related to BmMLV. Also similar to Bee macula-like virus (BeeMLV, NC_027631, synonym: Varroa destructor macula-like virus) that use bees and possibly also mites as a host, as demonstrated by sub-genomic RNA analyses (de Miranda et al., 2015). The maculavirus, Culex originated tymoviridae-like virus (CuTLV, NC_018703), exhibits cytopathic effects in mosquito cell lines (Wang et al., 2012). |
| *Osmia cornuta* nudivirus (OcNV) | dsDNA virus  *Nudiviridae*  unclassified | Ocor1  Obic3 | 100, 101, 150 | A nudivirus phylogenetically most related to Kallithea virus (KV; host *Drosophila melanogaster*) and *Oryctes rhinoceros* nudivirus (OrNV; host *Dynastinae* species). KV is not well studied, but OrNV has been studied since 1960s (Huger, 1966). Until present, OrNV is used as a biological control agent to combat rhinoceros beetle infestations in coconut and oil palm (Jackson, Crawford, & Glare, 2005). Huger demonstrated multiplication of OrNV in actively proliferating midgut cells of adult beetles by histological studies (Huger, 1972). |
| Sobemo-like virus | +ssRNA virus  unclassified | Ocor1 | 14, 100 | Putative RNA virus phylogenetically most related to unclassified insect-specific viruses and plant viruses in the unclassified genus *Sobemovirus* and families *Barnaviridae* and *Luteoviridae*. Highest RdRp similarity (63%) to Wenzhou sobemo-like virus 4 that is identified in mosquitoes (Shi et al., 2016). Also similar to viruses identified in sheep ticks (Pettersson et al., 2017), deer ticks (Tokarz et al., 2014), and fruit flies (Webster et al., 2015). |
| *Bombus cryptarum* densovirus | ssDNA virus  *Parvoviridae*  *Densovirinae*  unclassified | Bcry3 | 123, 127, 128, 142, 144 | A densovirus phylogenetically related to the genera *Ambidensovirus* and *Iteradensovirus,* and exhibiting an ambisense genome organization. The NS1 protein was most similar (36 % aa identity) to that of *Diaphorina citri* densovirus (Nouri, Salem, Nigg, & Falk, 2015) and less similar to the first reported Hymenopteran densovirus (28 % aa identity) from the red fire ant . All currently classified members of the genus *Ambidensovirus* are pathogenic to insect hosts of the orders Lepidoptera, Diptera, Orthoptera, Odonata, Hemiptera and Hymenoptera (Valles et al., 2013). A member of the genus *Iteradensovirus* adopts a mutualistic relation and protects its lepidopteran host against a baculovirus and a biopesticide (Xu, Liu, Graham, Wilson, & Wu, 2014). A member of the genus *Penstyldensovirus* has been reported to integrate into the host genome (Tang & Lightner, 2006). *Bombus cryptarum* densovirus forms together with Lone star tick densovirus 1 a separate group that awaits taxonomical classification. |
| *Bombus terrestris* densovirus | ssDNA virus  *Parvoviridae*  *Ambidensovirus* | Bter1 | 12 | Three fragmentary contigs, Bter1 _30546, Bter1 _3688, Bter1 _5589 (clcdenovo), with total length of 2111 nt were recovered that were highly similar (70-88 % nt identity, 60-74 % aa identity) to *Bombus cryptarum* densovirus. |
| *Bombus terrestris* nege-like virus | +ssRNA virus  unclassified | Bter1  Bter4 | 16, 18, 89 | A nege-like virus phylogenetically related to unclassified nege-like and virga-like viruses identified from invertebrates (Shi et al., 2016). RdRp most similar (36 % aa identity) to that of Xinzhou nematode virus 1 (NC_033728), identified in snake-associated nematodes in China. Less similar to Boutonnet virus of fruit flies (Webster, Longdon, Lewis, & Obbard, 2016) and negev-like viruses of mosquitoes from Australia (Shi et al., 2017). Most complete contig was DN5335_c0_g2_i1 (Bter4, trinity, 5759 nt). |
| *Bombus pascuorum* nege-like virus | +ssRNA virus  unclassified | Bpas1  Bpas3  Bpas4 | 6, 17, 65, 193 | Highly similar to *Bombus terrestris* nege-like virus (83 % nt identity, 92 % RdRp aa identity). Most complete contig was Bpas1_452 (clcdenovo, 5289 nt). |
| *Bombus cryptarum* nege-like virus | +ssRNA virus  unclassified | Bcry3 | ns | Three fragmentary contigs, Bcry3 _22286, Bcry3 _23320, Bcry3_27115 (clcdenovo), dissimilar to *Bombus terrestris* nege-like virus and *Bombus pascuorum* nege-like virus sequences. But similar (46 % aa identity) to a virga-like virus from mosquitoes (NC_033165) (Shi et al., 2016) and less similar to nege-like viruses of fruit flies (Webster et al., 2016). |
| *Osmia cornuta* nege-like virus | +ssRNA virus  unclassified | Ocor1  Ocor2  Ocor4 | 4, 13, 14, 100, 101, 28, 34, 35, 37, 74, 77, 79, 81 | Multiple fragmentary contigs in all *O. cornuta* libraries that were co-assembled to obtain a partial genome sequence of 3777 nt. This virus is phylogenetically more related to *Virgaviridae* than the nege-like viruses in *Bombus* sp and *Andrena haemorrhoa*. It is most similar (41 % RdRp aa identity) to Abisko virus (NC_035470), a virus identified in the moth *Epirrita autumnata* (de Miranda et al., 2017). |
| *Osmia bicornis* nege-like virus | +ssRNA virus  unclassified | Obic3 | ns | Highly similar to *Osmia cornuta* nege-like virus (95 % nt identity, 99 % RdRp aa identity). Seven fragmentary contigs with summed length 3678 nt. |
| *Andrena haemorrhoa* nege-like virus | +ssRNA virus  unclassified | Ahae2  Ahae3 | 157 | A nege-like virus phylogenetically related to members of the tentative genus *Sandewavirus* (Nunes et al., 2017). It is most similar (48 % RdRp aa identity) to Tanay virus (NC_024071), a virus isolated from mosquitoes (Nabeshima et al., 2014).  The partial genome sequence assembled from RNA-Seq reads is 8806 nt long and the sequence has been submitted to genbank accession MF998082. |
| *Andrena cineraria* nege-like virus | +ssRNA virus  unclassified | Acin4 | ns | Two short contigs, Acin4_29262 and Acin4_33131 (clcdenovo), similar to *Osmia cornuta* and *Osmia bicornis* nege-like viral sequences (73-75 % nt identity). Indeed, the contigs were also most similar (58 % aa identity) to Abisko virus (NC_035470). |
| *Andrena cineraria* rhabdovirus | -ssRNA virus  Mononegavirales  *Rhabdoviridae*  unclassified | Acin4 | 181, 187, 188, 189, 192 | Rhabdoviruses have a monopartite linear RNA genome encoding 5-6 proteins: N, P, M, G and L. Nine fragmentary contigs were manually assembled into a 6396 nt transcript with highest similarity to the L protein of rhabdovirus Wuhan Ant virus (NC_031276, 42 % RdRp aa identity) (Li et al., 2015). It was less similar (36% aa identity) to Apis rhabdovirus 1 (KY354231) (Remnant et al., 2017). Additionally, a single contig, Acin4_1645 (clcdenovo), was found by tblastn and rhabdoviral protein queries, and likely is the G protein transcript. Transcripts for N, P and M were not found. RT-PCR confirmed the presence of the virus in all 5 *A. cineraria* individuals. |
| *Osmia cornuta* toti-like virus (OcTV) | dsRNA virus  *Totiviridae* | Ocor1  Ocor2  Ocor4 | ns | A toti-like virus phylogenetically related to viral families *Totiviridae, Quadriviridae* and *Chrysoviridae,* but forming a separate clade with other toti-like viruses of a parasitoid wasp (Martinez, Lepetit, Ravallec, Fleury, & Varaldi, 2016), horseflies and water striders (Shi et al., 2016). Also similar to a recently characterized ant toti-like virus (Koyama et al., 2015). Blast also identified similar refseq records in solitary bees *Ceratina calcarata* and *Habropoda laboriosa* of the family *Apidae* indicating that toti-like viruses are present in other bees as well. The most complete contig of *O. cornuta* tot-like virus is Ocor2_2234 (clcdenovo) representing (+) transcript encoding |
| Narnavirus | ssRNA virus  *Narnaviridae*  *Narnavirus* | Aful1 | ns | Narnaviruses are small +ssRNA viruses naturally infecting fungi. Three contigs, Aful1_15268, Aful1_22034 and Aful1_29668 (clcdenovo), clustered in *Narnaviridae.* The sequences had similarity to Wenling narna-like virus 7 (NC_033049, 40 % RdRp aa similarity) identified from crustaceans (Shi et al., 2016) and Point-Douro narna-like virus from mosquitoes (Shi et al., 2017). |
| Cherry virus A (CVA) | +ssRNA virus  Tymovirales  *Betaflexiviridae*  *Capillovirus* | Bter1  Bter2  Bter4  Bpas1  Bpas2  Ocor1  Ocor2  Ocor4  Aful1 | ns | CVA has a monopartite, linear +ssRNA genome (NC_003689). CVA has a worldwide distribution and is found in various *Prunus* sp. among which *P. avium* (sweet cherry) and *P. domestica* (plum), although it has not yet been associated with a particular disease (Plantwise Knowledge Bank). To date, there is no known vector of this plant virus. Typically, multiple slightly different contigs were assembled in libraries, e.g. Ocor2_1199 was more similar to CVA isolate WK (LN879388) whereas Ocor2_1200 was more similar to CVA isolate 3137 13A1/13TF106_N6 (KY510897). |
| Cherry leaf roll virus (CLRV) | +ssRNA virus  Picornavirales  *Secoviridae*  Nepovirus | Bter1  Bcry3  Bter4  Bpas1  Bpas2  Ocor1  Ocor2  Obic3 | ns | CLRV has a bipartite, linear +ssRNA genome (NC_015414-15). CLRV or walnut ringspot virus is a subgroup C nepovirus comprising of multiple plant-specific strains that infect a wide range of wild and cultivated woody plant species. It imposes a serious threat to economically important trees including walnut and sweet cherry (SPHDS, 2016). CLRV has a worldwide distribution and virus particles are transmitted via grafting or through seeds and pollen. Here, contigs of both viral segments RNA1 and RNA2 were identified. Contigs were most similar to an unspecified isolates S84124-25 and the isolate Rube 74 (KU215413). |
| Prune dwarf virus (PDV) | +ssRNA virus  *Bromoviridae*  *Ilarvirus*  *Ilarvirus* subg. 4 | Aful1  Ocor4 | ns | PDV has a tripartite, linear +ssRNA genome (NC_008037-39). PDV or cherry ring mottle virus causes many disease variants in *Crataegus* and *Prunus* spp. It has a worldwide distribution and is like other ilarviruses pollen- and seedborne.  Typically, the third genomic segment RNA3 which encodes the movement and capsid proteins was most abundant. It was the only detected segment in library Ocor4. Segment RNA3 was not detected in library Aful1. Sequences from both libraries were most similar to PDV isolate 235C from *Prunus avium* (sweet cherry). |
| Prune virus F (PVF) | +ssRNA virus  Picornavirales  *Secoviridae*  *Fabavirus* | Bter2  Bter4  Bpas2  Ocor4 | ns | PVF has a bipartite, linear +ssRNA genome (KX269865, KX269871). PVF is recently described fabavirus infecting stone fruits. It has been associated with sweet cherry tree declines in the U.S.A. although its singular contribution to disease remains unkown (Villamor, Pillai, & Eastwell, 2017). Here, contigs of both viral segments RNA1 and RNA2 were identified in Bter2 (1 strain), Bter4 (2 strains), Bpas2 (1 strain) and Ocor4 (3 strains). |
| Strawberry latent ringspot virus (SLRSV) | +ssRNAvirus  Picornavirales  *Secoviridae*  Unassigned | Bpas4 Ocor4 | ns | SLRSV has a bipartite, linear +ssRNA genome (NC_006964-65). SLRSV or latent ring spot of strawberry virus  Infects many different crop species (Plantwise Knowledge Bank). A soil-borne plant nematode has been identified as a vector, although seed transmission also occurs. Here, SLRSV was associated with *Bombus pascuorum* and *Osmia cornuta* in a single locality (Torgny-Lamorteau). In both libraries, contigs of segments RNA1 and RNA2 were identified and the virus was averagely high abundant compared to other plant viruses. |
| Arabis mosaic virus (AMV) | +ssRNA virus  Picornavirales  *Secoviridae*  Nepovirus | Obic3 | ns | AMV has a bipartite, linear +ssRNA genome (NC_006056-57). AMV can infect many herbaceous and woody plants such as raspberry, strawberry rhubarb, *Prunus* spp., though infections are mostly latent (Plantwise Knowledge Bank). Here, an apparent single strain was identified associated with *Osmia bicornis.* Both segments RNA1 and RNA2 were covered by contigs. |
| Crimson clover cryptic virus 2 (CCCV2) | dsRNA virus  *Partitiviridae*  *Betapartitivirus* | Bpas4 | ns | CCCV2 has a bipartite, linear dsRNA genome (JX971982-83). CCCV2 is a cryptic virus that is commonly present in plants without causing disease (Lesker, Rabenstein, & Maiss, 2013). Here, CCCV2 was associated with *Bombus pascuorum* and contigs of only segment RNA2 were identified which encodes the coat protein. |

* RT-PCR +: the number represents the unique identifier that was given to each individual bee included in this study. The underscored line type corresponds to that of the RNA-Seq + library. ns = not screened.

**References**

Bailey, L., & Woods, R. D. (1977). Two more small RNA viruses from honey bees and further observations on sacbrood and acute bee-paralysis viruses. *Journal of General Virology, 37*, 175-182.

de Miranda, J. R., Cornman, R. S., Evans, J. D., Semberg, E., Haddad, N., Neumann, P., & Gauthier, L. (2015). Genome Characterization, Prevalence and Distribution of a Macula-Like Virus from Apis mellifera and Varroa destructor. *Viruses, 7*(7), 3586-3602. doi:10.3390/v7072789

de Miranda, J. R., Hedman, H. O., P., Stephan, J., Karlberg, O., Bylund, H., & Terenius, O. (2017). Characterization of a novel RNA virus discovered in the autumnal moth Epirrita autumnata in Sweden. *Viruses, 9*(8), 214.

Dheilly, N. M., Maure, F., Ravallec, M., Galinier, R., Doyon, J., Duval, D., . . . Mitta, G. (2015). Who is the puppet master? Replication of a parasitic wasp-associated virus correlates with host behaviour manipulation. *Proc Biol Sci, 282*(1803), 20142773. doi:10.1098/rspb.2014.2773

Huger, A. M. (1966). A virus disease of the Indian rhinoceros beetle, Oryctes rhinoceros(Linnaeus), caused by a new type of insect virus, Rhabdionvirus oryctes gen. n., sp. n. *J Invertebr Pathol, 8*(1), 38-51. Retrieved from <http://www.ncbi.nlm.nih.gov/pubmed/5905536>

Huger, A. M. (1972). Grundlagen zur biologischen Bekämpfung des Indischen Nashornkäfers, Oryctes rhinoceros (L.), mit Rhabdionvirus oryctes: Histopathologie der Virose bei Käfern. *Z Angew Entomol, 72*, 309-319.

Jackson, T. A., Crawford, A. M., & Glare, T. R. (2005). Oryctes virus--time for a new look at a useful biocontrol agent. *J Invertebr Pathol, 89*(1), 91-94. doi:10.1016/j.jip.2005.03.009

Katsuma, S., Tanaka, S., Omuro, N., Takabuchi, L., Daimon, T., Imanishi, S., . . . Shimada, T. (2005). Novel macula-like virus identified in Bombyx mori cultured cells. *Journal of Virology, 79*(9), 5577-5584. doi:10.1128/JVI.79.9.5577-5584.2005

Koyama, S., Urayama, S., Ohmatsu, T., Sassa, Y., Sakai, C., Takata, M., . . . Mizutani, T. (2015). Identification, characterization and full-length sequence analysis of a novel dsRNA virus isolated from the arboreal ant Camponotus yamaokai. *J Gen Virol, 96*(Pt 7), 1930-1937. doi:10.1099/vir.0.000126

Lesker, T., Rabenstein, F., & Maiss, E. (2013). Molecular characterization of five betacryptoviruses infecting four clover species and dill. *Arch Virol, 158*(9), 1943-1952. doi:10.1007/s00705-013-1691-x

Li, C. X., Shi, M., Tian, J. H., Lin, X. D., Kang, Y. J., Chen, L. J., . . . Zhang, Y. Z. (2015). Unprecedented genomic diversity of RNA viruses in arthropods reveals the ancestry of negative-sense RNA viruses. *Elife, 4*. doi:10.7554/eLife.05378

Martinez, J., Lepetit, D., Ravallec, M., Fleury, F., & Varaldi, J. (2016). Additional heritable virus in the parasitic wasp Leptopilina boulardi: prevalence, transmission and phenotypic effects. *J Gen Virol, 97*(2), 523-535. doi:10.1099/jgv.0.000360

Nabeshima, T., Inoue, S., Okamoto, K., Posadas-Herrera, G., Yu, F., Uchida, L., . . . Morita, K. (2014). Tanay virus, a new species of virus isolated from mosquitoes in the Philippines. *J Gen Virol, 95*(Pt 6), 1390-1395. doi:10.1099/vir.0.061887-0

Nouri, S., Salem, N., Nigg, J. C., & Falk, B. W. (2015). Diverse Array of New Viral Sequences Identified in Worldwide Populations of the Asian Citrus Psyllid (Diaphorina citri) Using Viral Metagenomics. *Journal of Virology, 90*(5), 2434-2445. doi:10.1128/JVI.02793-15

Nunes, M. R. T., Contreras-Gutierrez, M. A., Guzman, H., Martins, L. C., Barbirato, M. F., Savit, C., . . . Tesh, R. B. (2017). Genetic characterization, molecular epidemiology, and phylogenetic relationships of insect-specific viruses in the taxon Negevirus. *Virology, 504*, 152-167. doi:10.1016/j.virol.2017.01.022

Ongus, J. R., Peters, D., Bonmatin, J. M., Bengsch, E., Vlak, J. M., & van Oers, M. M. (2004). Complete sequence of a picorna-like virus of the genus Iflavirus replicating in the mite Varroa destructor. *J Gen Virol, 85*(Pt 12), 3747-3755. doi:10.1099/vir.0.80470-0

Peng, W. J., Li, J. L., Boncristiani, H., Strange, J. P., Hamilton, M., & Chen, Y. P. (2011). Host range expansion of honey bee Black Queen Cell Virus in the bumble bee, Bombus huntii. *Apidologie, 42*(5), 650-658. doi:10.1007/s13592-011-0061-5

Pettersson, J. H., Shi, M., Bohlin, J., Eldholm, V., Brynildsrud, O. B., Paulsen, K. M., . . . Holmes, E. C. (2017). Characterizing the virome of Ixodes ricinus ticks from northern Europe. *Sci Rep, 7*(1), 10870. doi:10.1038/s41598-017-11439-y

Ravoet, J., De Smet, L., Meeus, I., Smagghe, G., Wenseleers, T., & de Graaf, D. C. (2014). Widespread occurrence of honey bee pathogens in solitary bees. *J Invertebr Pathol, 122*, 55-58. doi:10.1016/j.jip.2014.08.007

Remnant, E. J., Shi, M., Buchmann, G., Blacquiere, T., Holmes, E. C., Beekman, M., & Ashe, A. (2017). A Diverse Range of Novel RNA Viruses in Geographically Distinct Honey Bee Populations. *Journal of Virology, 91*(16). doi:10.1128/JVI.00158-17

Schoonvaere, K., De Smet, L., Smagghe, G., Vierstraete, A., Braeckman, B. P., & de Graaf, D. C. (2016). Unbiased RNA Shotgun Metagenomics in Social and Solitary Wild Bees Detects Associations with Eukaryote Parasites and New Viruses. *PLoS One, 11*(12), e0168456. doi:10.1371/journal.pone.0168456

Shi, M., Lin, X. D., Tian, J. H., Chen, L. J., Chen, X., Li, C. X., . . . Zhang, Y. Z. (2016). Redefining the invertebrate RNA virosphere. *Nature*. doi:10.1038/nature20167

Shi, M., Neville, P., Nicholson, J., Eden, J. S., Imrie, A., & Holmes, E. C. (2017). High-Resolution Metatranscriptomics Reveals the Ecological Dynamics of Mosquito-Associated RNA Viruses in Western Australia. *Journal of Virology, 91*(17). doi:10.1128/JVI.00680-17

Singh, R., Levitt, A. L., Rajotte, E. G., Holmes, E. C., Ostiguy, N., Vanengelsdorp, D., . . . Cox-Foster, D. L. (2010). RNA Viruses in Hymenopteran Pollinators: Evidence of Inter-Taxa Virus Transmission via Pollen and Potential Impact on Non-Apis Hymenopteran Species. *PLoS One, 5*(12). doi:ARTN e14357

10.1371/journal.pone.0014357

SPHDS. (2016). *National Diagnostic Protocol for Cherry Leaf Roll Virus, CLRV (cherry and walnut strains)*. Retrieved from

Tang, K. F., & Lightner, D. V. (2006). Infectious hypodermal and hematopoietic necrosis virus (IHHNV)-related sequences in the genome of the black tiger prawn Penaeus monodon from Africa and Australia. *Virus Res, 118*(1-2), 185-191. doi:10.1016/j.virusres.2006.01.003

Tokarz, R., Williams, S. H., Sameroff, S., Sanchez Leon, M., Jain, K., & Lipkin, W. I. (2014). Virome analysis of Amblyomma americanum, Dermacentor variabilis, and Ixodes scapularis ticks reveals novel highly divergent vertebrate and invertebrate viruses. *Journal of Virology, 88*(19), 11480-11492. doi:10.1128/JVI.01858-14

Valles, S. M., Schoemaker, D., Wurm, Y., Strong, C. A., Varone, L., Becnel, J. J., & Shirk, P. D. (2013). Discovery and molecular characterization of an ambisense densovirus from South American populations of *Solenopsis invicta*. *Biological Control, 67*, 431-439.

Villamor, D. E., Pillai, S. S., & Eastwell, K. C. (2017). High throughput sequencing reveals a novel fabavirus infecting sweet cherry. *Arch Virol, 162*(3), 811-816. doi:10.1007/s00705-016-3141-z

Wang, L., Lv, X., Zhai, Y., Fu, S., Wang, D., Rayner, S., . . . Liang, G. (2012). Genomic characterization of a novel virus of the family Tymoviridae isolated from mosquitoes. *PLoS One, 7*(7), e39845. doi:10.1371/journal.pone.0039845

Webster, C. L., Longdon, B., Lewis, S. H., & Obbard, D. J. (2016). Twenty-Five New Viruses Associated with the Drosophilidae (Diptera). *Evol Bioinform Online, 12*(Suppl 2), 13-25. doi:10.4137/EBO.S39454

Webster, C. L., Waldron, F. M., Robertson, S., Crowson, D., Ferrari, G., Quintana, J. F., . . . Obbard, D. J. (2015). The Discovery, Distribution, and Evolution of Viruses Associated with Drosophila melanogaster. *PLoS Biol, 13*(7), e1002210. doi:10.1371/journal.pbio.1002210

Xu, P., Liu, Y., Graham, R. I., Wilson, K., & Wu, K. (2014). Densovirus is a mutualistic symbiont of a global crop pest (Helicoverpa armigera) and protects against a baculovirus and Bt biopesticide. *PLoS Pathog, 10*(10), e1004490. doi:10.1371/journal.ppat.1004490

Zioni, N., Soroker, V., & Chejanovsky, N. (2011). Replication of Varroa destructor virus 1 (VDV-1) and a Varroa destructor virus 1-deformed wing virus recombinant (VDV-1-DWV) in the head of the honey bee. *Virology, 417*(1), 106-112. doi:10.1016/j.virol.2011.05.009
