# Supplementary material for: Study of the Metatranscriptome of Eight Social and Solitary Wild Bee Species Reveals Novel Viruses and Bee Parasites
Source: Front Microbiol. 2018 Feb 14;9:177. doi: 10.3389/fmicb.2018.00177 (PMC5817871; doi:10.3389/fmicb.2018.00177)
Supplement: Supplementary file 3 [file Table_2.DOCX]

Table S2. Description of protozoan and metazoan parasites associated with wild bees.

CLC/Trinity contigs are available from <https://figshare.com/collections/Study_of_the_virome_of_8_wild_bee_species_in_Belgium/3902704>

| Name | Current Taxonomy | RNA-Seq + | RT-PCR +* | Description |
| --- | --- | --- | --- | --- |
| *Crithidia bombi* | Flagellata  Kinetoplastida  *Trypanosomatidae* | Bter1  Bter2  Bter4  Bpas1  Bpas2  Bpas4  *Ocor4* | 8, 32, 86, 1, 24, 26, 30, 91, *74*, *78*, *81*, | One of two monoxenous, trypanosome parasites of bumble bees (Lipa & Triggiani, 1988), other is *C. expoeki* (R. Schmid-Hempel & Tognazzo, 2010), phylogenetically related to *Crithidia mellificae* and *Lotmaria passim* of *Apis mellifera*. *C. bombi* lowers the fitness of hibernating queens by 40 % (Brown, Schmid-Hempel, & Schmid-Hempel, 2003) and thus affects colony success and reproduction. The prevalence of this parasite in *B. terrestris* queens has been reported to be as high as 47.5 % (Shykoff & Schmid-Hempel, 1991). In earlier work, we detected *C. bombi* in solitary bees (Ravoet et al., 2014; Schoonvaere et al., 2016) and the presence here of a distinct parasite transcriptome in *O. cornuta* indicates that it establishes infestations comparable to those observed in bumble bees. The *C. bombi* transcriptome was represented by 1655 contigs of the Ocor4 library. |
| *Crithidia pragensis* | Flagellata  Kinetoplastida  *Trypanosomatidae* | Ocor1 | 100 | Originally characterized in the fly *Cordilura albipes* (Diptera: *Scathophagidae*) at the molecular and ultrastructure level (Yurchenko et al., 2014). From the parasite transcriptome, we found that the Cp-GAPDH transcript (Ocor1_contig_9703, clcdenovo) was identical to that of *C. pragensis* (KF482060). The sequence had 99 % nt identity (827/828 identities) to *C. pragensis* but the apparently dissimilar nucleotide was in fact a degenerate base S (G or C) in the reference sequence (C in Ocor1_contig_9703). Interestingly, the authors who described *C. pragensis (Yurchenko et al., 2014)* also found viral particles in the mitochondrial lumen of this parasite by ultrastructural data and they associated this observation with dsRNA viruses of the family *Totiviridae.* Whether the toti-like viruses detected in *O. cornuta* and other bees are associated with trypanosome infestation remains to be investigated. The Ocor1 library counted 291 contigs that correspond to this parasite’s transcriptome. |
| *Nosema bombi* | Fungi  Microsporidia  *Nosematidae* | Bcry3  Bpas3 | 123, 63 | An obligate intracellular parasite of bumble bees that is phylogenetically most related to *N. apis* and *N. neumanni,* followed by *N. thomsoni, N. portugal* and *N. ceranae.* The primary site of spore development are the Malpighian tubules (Mcivor & Malone, 1995), but extends secondarily to other tissues including midgut and fat bodies (Macfarlane, Lipa, & Liu, 1995). *N. bombi* infestation negatively impacts the colony founding capability of queens (Mcivor & Malone, 1995; van der Steen, 2008) and thus affects reproduction. The prevalence of *N. bombi* in spring queens ranges from 0 to 100 % (Brown, 2017; Macfarlane et al., 1995). Here, 1018 contigs of library Bcry3 and 862 contigs of library Bpas3 clustered in Microsporidia (or taxa below) and represent the parasite’s transcriptome. |
| *Nosema thomsoni* | Fungi  Microsporidia  *Nosematidae* | Ahae2 | 157, 155, 172 | Originally characterized in the moth *Choristoneura conflictana* (Lepidoptera: *Tortricidae*) (Wilson & Burke, 1971), and later close relatives to *N. thomsoni* were reported in *Harmonia axyridis* (Vilcinskas, Stoecker, Schmidtberg, Rohrich, & Vogel, 2013) and *Andrena vaga* (Ravoet et al., 2014). *N. thomsoni* is phylogenetically more related to *N. ceranae* than to *N. bombi*. Here, 620 contigs of the Ahae2 library clustered in Microsporidia (or taxa below) and represent the parasite’s transcriptome. |
| *Tubulinosema* sp. | Microsporidia  *Tubulinosematidae* | Bpas2 | 24 | Members of the family *Tubulinosematidae* are obligate intracellular parasites of insects but infections are reported in immunosuppressed human patients (Choudhary et al., 2011). *Tubulinosema* parasites infect various insect tissues including fat body, midgut, Malpighian tubules and muscles (Malysh et al., 2013). *Tubulinosema pampeana* was recently described from the bumble bee *Bombus atratus* in Argentina (Plischuk, Sanscrainte, Becnel, Estep, & Lange, 2015). This parasite mainly infected fat bodies and had a low average prevalence (5.3 %) although higher in queens (10.2 %). Here, 176 contigs of library Bpas2 clustered in Microsporidia (predominantly Tubulinosematidae). |
| *Apicystis* sp. | Alveolata  Apicomplexa  Neogregarinorida | Bpas1  Bpas4 | 6, 88 | A neogregarine parasite closely related to *Apicystis bombi* (Lipa & Triggiani, 1996) and *Mattesia* spp (Valles & Pereira, 2003), though more similar to *A. bombi* based on sporocyst morphology. *A. bombi* has been reported to infect *Apis mellifera* and *Bombus* spp. (Lipa & Triggiani, 1996). The primary sites where infection takes place are the visceral and parietal fat body tissues. Due to the high degree of similarity to *A. bombi* sporocyst morphology, *A. bombi* and *Apicystis* sp. have likely been considered as one and the same species leading to inaccurate prevalence numbers. Here, 1192 contigs of library Bpas1 and 1700 contigs of library Bpas4 clustered in Alveolata and represent the transcriptome of *Apicystis* sp. Considerable nucleotide dissimilarity between *Apicystis* sp. and *A. bombi* transcripts (parts of the *A. bombi* transcriptome was obtained in a previous study (Schoonvaere et al., 2016)) supports the classification of *Apicystis* sp. as a separate species. For example, the partial GAPDH transcripts had 740 of 996 (74 %) nt identities and the partial RPS8 transcripts had 469/655 (72 %) nt identities. |
| *Locustacarus buchneri* | Arthropoda  Acariformes *Podapolipidae* | Bpas1  Bpas3 | 1, 108, 190(bpas4) | An internal parasitic mite of bumble bees residing in tracheae and air sacs (Husband & Shina, 1970; Stammer, 1951). The parasite has been reported in Europe, North America, New Zealand, South America and Japan (Goka, Okabe, Yoneda, & Niwa, 2001; Macfarlane et al., 1995; Plischuk, Pocco, & Lange, 2013). Prevalence numbers vary greatly, the highest reported in N America (24 %) (Goldblatt & Fell, 1984). Global trade of commercial bumble bee colonies facilitates the spread of *L. buchneri* to new localities (Rozej et al., 2012). Here, RNA-Seq evidence of *L. buchneri* was scarce, only few one or two contigs in each library. |
| *Sphaerularia bombi* | Nematoda  Tylenchida  *Sphaerulariidae* | Bter2  Bpas2 | 32, 24, 26 | An internal parasitic nematode of bumble bees that infects (and sterilize) queens (Pionar & Van der Laan, 1972). Here, 9120 contigs of library Bter2 and 4872 contigs of library Bpas2 clustered in Nematoda and represent the transcriptome of *S. bombi*. |
| *Myopa testacea* | Arthropoda  Diptera  *Conopidae* | Ocor1  Acin4 | 101, 187, 189, 192 | Dipterans of the family *Conopidae* are internal parasites of mainly aculeate Hymenoptera. Larvae always develop in the abdomen (typically one larva per bee) and consume most of the host internal organs. Prevalence numbers are generally recorded for bumble bee workers and range from 8 to over 60 % (Macfarlane et al., 1995; Shykoff & Schmid-Hempel, 1991; Smith, 1969). The life expectancy of conopid infested bumble bees is halved as well as colony development and queen production rates (Macfarlane & Pengelly, 1975; P. Schmid-Hempel & Schmid-Hempel, 1988). *Myopa* sp. are commonly found in solitary bees (Smith, 1966). Here, 4947 contigs of library Ocor1 and 2750 contigs of library Acin4 represented the parasite’s transcriptome (COI: Ocor1_contig_1771, Acin4_contig_1759). |
| *Myopa tesselatipennis / hirsuta* | Arthropoda  Diptera  *Conopidae* | Ahae2 | 157, 155 | Discrimination between *M. tesselatipennis* and *M.hirsuta* could not be made based on COI barcode gene as both species were among top hits (100 % similarity) by BOLD systems identification (Ratnasingham & Hebert, 2007). Here, 3831 contigs of library Ahae2 represented the parasite’s transcriptome (COI: Ahae2_contig_2977). |

* RT-PCR +: the number represents the unique identifier that was given to each individual bee included in this study. The underscored line type corresponds to that of the RNA-Seq + library. ns = not screened.

**References**

Brown, M. J. F. (2017). Microsporidia: An Emerging Threat to Bumblebees? *Trends Parasitol*. doi:10.1016/j.pt.2017.06.001

Brown, M. J. F., Schmid-Hempel, R., & Schmid-Hempel, P. (2003). Strong context-dependent virulence in a host-parasite system: reconciling genetic evidence with theory. *Journal of Animal Ecology, 72*(6), 994-1002. doi:DOI 10.1046/j.1365-2656.2003.00770.x

Choudhary, M. M., Metcalfe, M. G., Arrambide, K., Bern, C., Visvesvara, G. S., Pieniazek, N. J., . . . Saeed, M. U. (2011). Tubulinosema sp. microsporidian myositis in immunosuppressed patient. *Emerg Infect Dis, 17*(9), 1727-1730. doi:10.3201/eid1709.101926

Goka, K., Okabe, K., Yoneda, M., & Niwa, S. (2001). Bumblebee commercialization will cause worldwide migration of parasitic mites. *Mol Ecol, 10*(8), 2095-2099. Retrieved from <http://www.ncbi.nlm.nih.gov/pubmed/11555253>

Goldblatt, J. W., & Fell, R. D. (1984). Parasites and parasitization rates in bumble bee queens, *Bombus* spp. (Hymenoptera: Apidae), in Southwestern Virginia. *Environmental Entomology, 13*(1661-1665).

Husband, R. W., & Shina, R. N. (1970). A revision of the genus *Locustacarus* with a key to the genera of the family Podapolipidae (Acarina). *Annals of the Entomological Society of America, 63*, 1152-1162.

Lipa, J. J., & Triggiani, O. (1988). Crithidia-Bombi Sp N. A Flagellated Parasite of a Bumblebee Bombus-Terrestris L (Hymenoptera, Apidae). *Acta Protozoologica, 27*(3-4), 287-&. Retrieved from <Go to ISI>://WOS:A1988T736500009

Lipa, J. J., & Triggiani, O. (1996). Apicystis gen nov and Apicystis bombi (Liu, Macfarlane & Pengelly) comb nov (Protozoa: Neogregarinida), a cosmopolitan parasite of Bombus and Apis (Hymenoptera: Apidae). *Apidologie, 27*(1), 29-34. doi:DOI 10.1051/apido:19960104

Macfarlane, R. P., Lipa, J. J., & Liu, H. J. (1995). Bumble bee pathogens and internal enemies. *Bee World, 76*(3), 130-148. Retrieved from <Go to ISI>://WOS:A1995RW87100004

Macfarlane, R. P., & Pengelly, D. H. (1975). Conopidae and Sarcophagidae (Diptera) as parasites of adult Bombinae (Hymenoptera) in Ontario. *Proceedings of the Entomological Society of Ontario, 165*, 55-59.

Malysh, J. M., Tokarev, Y. S., Sitnicova, N. V., Martemyanov, V. V., Frolov, A. N., & Issi, I. V. (2013). *Tubulinosema loxostegi* sp. n. (Microsporidia: Tubulinosematidae) from the Beet Webworm *Loxostege sticticalis* L. (Lepidoptera: Crambidae) in Western Siberia. *Acta Protozoologica, 52*(4). doi:DOI 10.4467/16890027AP.13.028.1319

Mcivor, C. A., & Malone, L. A. (1995). *Nosema bombi*, a Microsporidian Pathogen of the Bumble Bee *Bombus terrestris* (L). *New Zealand Journal of Zoology, 22*(1), 25-31. Retrieved from <Go to ISI>://WOS:A1995RQ16300003

Pionar, G. O., & Van der Laan, P. A. (1972). Morphology and life history of *Sphaerularia bombi*. *Nematologica, 18*(2), 239-252. doi:10.1163/187529272X00476

Plischuk, S., Pocco, M. E., & Lange, C. E. (2013). The tracheal mite Locustacarus buchneri in South American native bumble bees (Hymenoptera: Apidae). *Parasitol Int, 62*(6), 505-507. doi:10.1016/j.parint.2013.07.006

Plischuk, S., Sanscrainte, N. D., Becnel, J. J., Estep, A. S., & Lange, C. E. (2015). Tubulinosema pampeana sp. n. (Microsporidia, Tubulinosematidae), a pathogen of the South American bumble bee Bombus atratus. *J Invertebr Pathol, 126*, 31-42. doi:10.1016/j.jip.2015.01.006

Ratnasingham, S., & Hebert, P. D. N. (2007). BOLD: The Barcode of Life Data System ([www.barcodinglife.org)](http://www.barcodinglife.org)). *Molecular Ecology Notes, 7*(3), 355-364. doi:10.1111/j.1471-8286.2006.01678.x

Ravoet, J., De Smet, L., Meeus, I., Smagghe, G., Wenseleers, T., & de Graaf, D. C. (2014). Widespread occurrence of honey bee pathogens in solitary bees. *J Invertebr Pathol, 122*, 55-58. doi:10.1016/j.jip.2014.08.007

Rozej, E., Witalinski, W., Szentgyorgyi, H., Wantuch, M., Moron, D., & Woyciechowski, M. (2012). Mite species inhabiting commercial bumblebee (Bombus terrestris) nests in Polish greenhouses. *Exp Appl Acarol, 56*(3), 271-282. doi:10.1007/s10493-012-9510-8

Schmid-Hempel, P., & Schmid-Hempel, R. (1988). Parasitic flies (Conopidae, Diptera) may be important stress factors for the ergonomics of their bumblebee host. *Ecol. Entomology, 13*, 469-472.

Schmid-Hempel, R., & Tognazzo, M. (2010). Molecular Divergence Defines Two Distinct Lineages of Crithidia bombi (Trypanosomatidae), Parasites of Bumblebees. *Journal of Eukaryotic Microbiology, 57*(4), 337-345. doi:10.1111/j.1550-7408.2010.00480.x

Schoonvaere, K., De Smet, L., Smagghe, G., Vierstraete, A., Braeckman, B. P., & de Graaf, D. C. (2016). Unbiased RNA Shotgun Metagenomics in Social and Solitary Wild Bees Detects Associations with Eukaryote Parasites and New Viruses. *PLoS One, 11*(12), e0168456. doi:10.1371/journal.pone.0168456

Shykoff, J., & Schmid-Hempel, P. (1991). Incidence and effects of four parasites in natural populations of bumble bees in Switzerland. *Apidologie, 22*, 117-125.

Smith, K. G. V. (1966). The larva of *Thecophora occidensis*, with comments upon the biology of Conopidae (Diptera. *J. Zool. Lond., 149*, 263-276.

Smith, K. G. V. (1969). *The identification of Britisch insects, Vol. X. Part 3 (a) Diptera, Conopidae*. London: Royal Entomological Society of London.

Stammer, H. J. (1951). Eine neue Tracheenmilbe, *Bombacarus buchneri* n.g.n.sp. (Acar., Podapolipodidae). *Zool. Anz., 146*, 137-150.

Valles, S. M., & Pereira, R. M. (2003). Use of ribosomal DNA sequence data to characterize and detect a neogregarine pathogen of Solenopsis invicta (Hymenoptera: Formicidae). *J Invertebr Pathol, 84*(2), 114-118. Retrieved from <http://www.ncbi.nlm.nih.gov/pubmed/14615220>

van der Steen, J. J. M. (2008). Infection and transmission of *Nosema bombi* in *Bombus terrestris* colonies and its effect on hibernation, mating and colony founding. *Apidologie, 39*(2), 273-282.

Vilcinskas, A., Stoecker, K., Schmidtberg, H., Rohrich, C. R., & Vogel, H. (2013). Invasive harlequin ladybird carries biological weapons against native competitors. *Science, 340*(6134), 862-863. doi:10.1126/science.1234032

Wilson, G. G., & Burke, J. M. (1971). Nosema thomsoni n. sp., a microsporidian from Choristoneura conflictana (Lepidoptera: Tortricidae). *Can J Zool, 49*(5), 786-788. Retrieved from <http://www.ncbi.nlm.nih.gov/pubmed/5557911>

Yurchenko, V., Votypka, J., Tesarova, M., Klepetkova, H., Kraeva, N., Jirku, M., & Lukes, J. (2014). Ultrastructure and molecular phylogeny of four new species of monoxenous trypanosomatids from flies (Diptera: Brachycera) with redefinition of the genus Wallaceina. *Folia Parasitol (Praha), 61*(2), 97-112. Retrieved from <http://www.ncbi.nlm.nih.gov/pubmed/24822316>
