# Supplementary material for: Study of the Metatranscriptome of Eight Social and Solitary Wild Bee Species Reveals Novel Viruses and Bee Parasites
Source: Front Microbiol. 2018 Feb 14;9:177. doi: 10.3389/fmicb.2018.00177 (PMC5817871; doi:10.3389/fmicb.2018.00177)
Supplement: Supplementary file 4 [file Data_Sheet_1.DOCX]

Supplementary information for Materials & Methods

A glimpse of the virome of eight social and solitary wild bee species

Karel Schoonvaere*, Guy Smagghe, Frédéric Francis, Dirk C. de Graaf

*** Correspondence:** Karel Schoonvaere: karel.schoonvaere@gmail.com

# Species determination by COI barcode sequencing.

DNA was extracted from a single middle bee leg by the Chelex method [1]. Tibia, femur and basal tarsa were cut longitudinally using a sterile n°11 razor blade and incubated at 55°C for 16 hours and 1000 rpm (Esco) in 200 µl 5 % chelex solution and 1 mg/ml proteinase K. Digestion was ended by incubating samples at 95° for 15 min. Samples were centrifuged at 14k rpm for 5 min and the supernatans was collected. This step was repeated once and samples were stored at -20°C for later molecular analysis. The COI amplicon was sequenced using Platinum Pfx DNA Polymerase (Invitrogen). Primer sequences were forward LCO1490-modified (5'-TATCAACCAATCATAAAGATATTGG) and reversed HCO2198 (5'-TAAACTTCAGGGTGACCAAAAAATCA). Twenty-five µl PCR reaction mixes contained 1 µl undiluted chelex DNA, 10x Pfx buffer, 1 mM MgSO_4_, 0.2 mM dNTPs, 2 µM LCO1490-modified, 2 µM HCO2198, 1 U Pfx and ddH_2_O. PCR settings were 5 min @ 95 °C, followed by 35 cycles of (15 s @ 94 °C, 30 s @ 50 °C and 45 s @ 68 °C), final elongation 10 min @ 68°C, and held by 4°C. PCR products for Sanger sequencing were diluted 4 times (5 µl PCR product + 15 µl ddH_2_O). Primers for Sanger sequencing were diluted to 10 µM. Amplicons were sequenced by the Sanger method in both orientations using LCO1490-modified and HCO2198 primers at GATC Biotech (Constance, Germany). A 658 bp consensus excluding the primer sequences was generated using SeqTrace v0.9.0 [2]. The consensus sequences were queried to the BOLD Systems [3] for species confirmation. A subset of five individuals of the same BOLD-confirmed species was selected for further analysis. In case of *Andrena fulva* and *Andrena cineraria* the primer pair picked up the endosymbiont Wolbachia instead of the host COI gene which is a known issue to occur [4]. However, since both *A. fulva* and *A. cineraria* are not cryptic bee species in Belgium we were confident enough to include them into NGS without COI barcode confirmation. Later the species uniqueness was confirmed by subjecting the assembled COI contig to BOLD (Acin: 98.3 %, Aful: 100 %).

# Illumina transcriptome sequencing (outsourced, Genohub).

The quantity and quality of the extracted RNA samples were assessed on the NanoDrop 2000c and Agilent TapeStation and sequencing libraries were constructed using the Illumina TruSeq Stranded mRNA Library Preparation kit following manufacturer’s instructions. The RNA was diluted to 10 ng/µL in nuclease-free ultra-pure water and 500 ng was subjected to two rounds of mRNA selection with poly-T oligo-attached magnetic beads. Captured RNA was then fragmented to 200-250 bp length, converted to cDNA and subjected to blunt ending and adapter ligation. This was followed by PCR to selectively enrich for DNA fragments that have adapter molecules on both ends. The resulting sequencing library was then quantified on the Agilent TapeStation using the high sensitive D1000 Tape. Finally, sequencing libraries were loaded onto a single lane of the Illumina HiSeq 2500 System and run in rapid run mode to produce an average of 20 million reads per sample in the paired-end 2x100 base read format.

# Bio-informatics analysis.

## Quality assessment and *in silico* de novo assembly of transcripts

Raw sequence reads were quality assessed using FastQC software [5]. Reads were clipped at their 5'end (12 nt) and 3' end (3 nt) and subsequently trimmed based according to base quality (quality limit > 0.05) using CLC de novo Trim sequences tool (Qiagen). An additional adapter trimming step was included (TruSeq Adapter Index 7; Illumina PCR primer). Only high-quality, surviving paired-end reads longer than 50 bp were retained. Across the 16 libraries, an average of 97.9 % of the raw NGS reads were retained. High-quality reads were assembled into contigs using CLC de novo assembler (default parameters) within the CLC Genomics Workbench v8.5 (Qiagen) software and Trinity v2.2.0 [6] (default parameters + get_longest_isoform_seq_per_trinity_gene.pl). In both sets, only contigs longer than 200 bp were retained. CLC generated on average 37082 contigs and Trinity generated on average 45678 contigs.

## Annotation

CLC de novo contigs were annotated by the blastn command (parameters: evalue 1e-6, num_aligments 20, num_descriptions 20, wordsize 11, penalty -3, reward 2, gapopen 5, gapextend 2, default output format) within the BLAST+ v2.2.31 utilities [7]. Files were split processed by GNU parallel [8] on a computing grid (192 CPUs at a time) to speed up analysis time. The database used for blastn was the nt database downloaded on 2 August 2016 from the NCBI FTP portal. CLC de novo contigs were annotated by the diamond blastx command (parameters: -e 1e-5, -k 25, --sensitive) within the DIAMOND v0.8.17 software [9]. The database used for diamond blastx was the nr database downloaded on 2 August 2016 from the NCBI FTP portal. Raw .daa files were converted to .sam files using the diamond view command to facilitate downstream analysis.

## Metagenomics clustering

Annotated files were separately imported into MEGAN6 software [10] for metagenomics clustering. The mapping file used for blastn annotations was nucl_gi2tax-Aug2016.bin and parameters were MinScore 80, MaxExpected 0.01, TopPercent 5, MinSupport 5, mode BlastN. The mapping file used for diamond blastx annotations was prot_gi2tax-Aug2016X.bin and parameters were MinScore 50, MaxExpected 0.01, TopPercent 5, MinSupport 5, mode BlastX. Contigs were summarized at the level of well-supported taxa (Bactera, Alveolata, Euglenozoa, Dikarya, Microsporidia, Nematoda, Chelicerata, Diptera, Hymenoptera, Viridiplantae, Viruses) and fasta sequences were extracted from the leaves. A custom bash script was written to merge the taxon-binned multifasta files from blastn (nucleotide) and diamond blastx (protein) annotations. We collected and manually sorted the few contigs that had a dubious taxon assignment. We also refined the resolution of the taxon Viruses in order to obtain a better resolution to the species (e.g. Bee macula-like virus 2) or group (e.g. negative-sense single-stranded RNA viruses) level. This resulted in 30 informative taxa.

## Abundance analysis

High-quality forward ("left") reads were mapped to the taxon-binned contigs using HISAT2 v2.0.4 [11] and parameters --score-min L,0.0,-0.2 and --mp 6,2 (this parameter combination generally allowed at most 3 mismatches in a 85 nt read and at most 2 mismatches in a 50 nt read). Per taxon per NGS library, the total number of aligned reads (unique + multimapping reads) was gathered into the table "counts", the total reference length into the table "lengths" and the number of positive individuals in the library pool of 5 individuals into the table "pcr.csv". The three tables were imported into R v3.3.0 [12]. The normalized (average) coverage was calculated as follows:

$$C_{N,taxon}= \log_{10} \left[ \frac{\mathrm{counts}_{taxon}}{\mathrm{lengths}_{taxon}*\left( \frac{\mathrm{counts}_{hymenoptera}}{\mathrm{lengths}_{hymenoptera}} \right)} \right]$$

where *taxon* is one of the 30 well-supported taxa or refined virus species/group by MEGAN6 metagenomics analysis. The denominator includes a within-library normalization factor that is equal to the host average coverage in that library. The values were rescaled so that the C_N_ of the host taxon *hymenoptera* equals 1. Thus, a C_N_ > 1 represents a taxon with higher average coverage than the host whereas a C_N_ < 1 represents a taxon with lower average coverage than the host. Taxa that were not supported across the 16 libraries were given a *NA* value. The resulting 16 x 30 abundance matrix containing C_N_ and *NA* values was visualized as a heatmap using ggplot2 v2.2.1 [13]. The R script along with other scripts and datasets are available from the figshare collection at https://figshare.com/collections/Study_of_the_virome_of_8_wild_bee_species_in_Belgium/3902704

# Molecular assays

## Primer look-up table

| **Target** | Primer name | Primer s**equence (5'-3')** | **Tm** | **Ta** | **size (bp)** | **Reference** |
| --- | --- | --- | --- | --- | --- | --- |
| Crithidia bombi | KS62_cbmandh-f | CAAGCCATTCAGCTTGTTCA | 60 | 55 | 463 | This study |
| SSU | KS63_cbmandh-r | GGAGATTATCGGCAGCATGT | 60,1 | 55 |  | This study |
| Crithidia pragensis | CB-SSUrRNA-F2 | CTTTTGACGAACAACTGCCCTATC | 60.4 | 55 | 727 | Ravoet, unpublished |
| SSU | KS35_cp-ssu-r1 | GCCGAAGCCCAAGCCAAG | 61.8 | 55 |  | This study |
| Neogregarine | KS46_NeoSSUf | CAACCCCATGCAAGTATCAA | 59,4 | 55 | 166 | This study |
| SSU | KS47_NbpSSUr | TGCCAATAAAAACAAAGCTCAA | 59,8 | 55 |  | This study |
| Locustacarus buchneri | KS43_Lbco1f | CCCACTATCAAGAATTCCATTCC | 56,97 | 55 | 530 | This study |
| CO1 | KS44_Lbco1r | GCTGCTGTGAAGTATGCTCG | 59,35 | 55 |  | This study |
| Sphaerularia bombi | KS39_sbGAPDHf | ATCGGTGACAAAGTCGGTGG | 60,32 | 58 | 710 | This study |
| GAPDH | KS40_sbGAPDHr | GCGTTAAGGCCGAGAACAAC | 59,83 | 58 |  | This study |
| Myopa spp. | KS77_myopaCOIf | GGTGGATCTTCTGTTGATTTAGC | 59,1 | 55 | 751 | This study |
| COI | KS78_myopaCOIr | TTGCAAAAACAGCACCCATA | 60,1 | 55 |  | This study |
| Nosema bombi | qNoUF2 | GGATTGTGCGGCTTAATTTGACTC | 66,87 | 60 | 80 | qPCR, unpublished |
| SSU | qNoBR | ATTCTCGAATCAGGATTCTCTCAGAA | 61,58 | 60 |  | qPCR, unpublished |
| Nosema thomsoni | KS59_nost-f | ACTTCCCCCTTGTGGCTTAC | 60,4 | 55 | 296 | This study |
| SSU | KS60_nost-r | TTCCAAGACAGTGGGTTTCC | 59,9 | 55 |  | This study |
| Tubulinosema sp. | KS145_tubnF1 | CTTGAATCAGAGGAAAGGAGGACT | 61,4 | 55 | 682 | This study |
|  | KS146_tubnR1 | AATTACAAAGGATGATCCCAGTAGC | 60,9 | 55 |  | This study |
| Metschnikowia sp. | KS165_mets101f | CCCTTAATCGGGCCATAGTT | 60,16 | 55 | 232 | This study |
|  | KS166_mets332r | AACATCCTTGGCAAATGCTT | 59,57 | 55 |  | This study |
| Osmia nudivirus (ONV) | KS41_NVpif2f | TACGTTTGCGCCTTTCGATG | 59,56 | 55 | 534 | This study |
| pif-2 | KS42_NVpif2r | TTCGATCCGTTGACCACCAC | 60,32 | 55 |  | This study |
| Varroa destructor virus 1 | VDV1-F1409 | GCCCTGTTCAAGAACATG | 54,9 | 50 | 413 | [14] |
|  | DWV-B1806 | CTTTTCTAATTCAACTTCACC | 51 | 50 |  | [14] |
| BQCV | BQCV700-F | TGGTCAGCTCCCACTACCTTAAAC | 58,4 | 55 | 678 | [15] |
|  | BQCV700-R | GCAACAAGAAGAAACGTAAACCAC | 55,1 | 55 |  | [15] |
| A. haemorrhoa iflavirus | KS50_ahifla-f | TTTCCCGTGAAGACAGAACC | 60,1 | 55 | 411 | This study |
|  | KS51_ahifla-r | AGTCTTCCCATCCAGAGCAA | 59,8 | 55 |  | This study |
| B. terrestris densovirus | KS155_btdenso-f | ACCAAGATCATTGGCTCCAC | 59,9 | 55 | 449 | This study |
|  | KS156_btdenso-r | TGCAATGTGCATCATCTTCTT | 59,3 | 55 |  | This study |
| B. cryptarum densovirus | KS157_bcdenso-f | TTACCTCCAGCTCCGATGAC | 60,2 | 55 | 487 | This study |
|  | KS158_bcdenso-r | CTGGTGAAACGAAACAAGCA | 59,9 | 55 |  | This study |
| B. terrestris negevirus | KS149_btnege-f | GTGATGCGCGGAACTTTATT | 60,1 | 55 | 404 | This study |
|  | KS150_btnege-r | GACAGACGTCGCATCAAGAA | 60 | 55 |  | This study |
| B. pascuorum negevirus | KS151_bpnege-f | TACTGGATGGGAGCCTTTTG | 60,1 | 55 | 451 | This study |
|  | KS152_bpnege-r | CCTGTACGCAACGAGCTGTA | 60,1 | 55 |  | This study |
| O. cornuta negevrius | KS153_ocnege-f | CATCAATGCCAGCGAGTAGA | 60 | 55 | 498 | This study |
|  | KS154_ocnege-r | GCATTGTCATTTCCGTGTTG | 60 | 55 |  | This study |
| A. haemorrhoa negevirus | KS147_ahnege-f | TATATGTTTGCCGGTGACGA | 60 | 55 | 489 | This study |
|  | KS148_ahnege-r | TACAAGGTCACGACGACCAA | 60,2 | 55 |  | This study |
| SRBV | KS118-chu774f | AACGTCTCGAGTTGTGAAGTACA | 59 | 55 | 774 | This study |
|  | KS119-chu774r | CTCACCCTCTGGCCTCAGT | 60,4 | 55 |  | This study |
| GABV | KS29-WCVf2/pha782f | CTCATATGCACCCTGCTCCAA | 63,4 | 55 | 782 | This study |
|  | KS30-WCVR2/pha782r | GGTGGCCTCAAGGAATGTTGA | 64,5 | 55 |  | This study |
| Maculavirus | KS48_macula-f | CCTGCTTCTCTTGGTTTTGC | 60 | 55 | 486 | This study |
|  | KS49_macula-r | AGCGTTTTGCTCGAGTTGAT | 60 | 55 |  | This study |
| Sobemovirus | KS159_ocsobemo-f | CAGGGCTTGGTTACAGCAG | 59,5 | 55 | 454 | This study |
|  | KS160_ocsobemo-r | CGCTAGCTTCCTGGTTCTTG | 60,1 | 55 |  | This study |
| A. cineraria rhabdovirus | KS161_acrhabdo-f | GCCGAGCATGTTTTATCGTT | 60,1 | 55 | 423 | This study |
|  | KS162_acrhabdo-r | AAGGTTCTCTGCCGCTAACA | 60 | 55 |  | This study |
| Tubulinosema (ExpandLT) | KS32_ssu18f_µs | CACCGCAGATTGATTCTGTC | 59.2 | 50 | 1913 | Modified from [16] |
|  | KS33_lsu580r_µs | GGTCCGTGTTTCAAGACGG | 56.2 | 50 |  | [16] |
| NeoL (Pfx) | ApUF2 | ATCTGGTTGATCCTGCCAGT | 58.7 | 55 | 1067 | [17] |
|  | ApBR1 | TGAAAGCGGCGTATACATGA | 57.4 | 55 |  | [17] |
| NeoR (Pfx) | ApBF1 | CGTACTGCCCTGAATACTCCAG | 60.2 | 55 | 972 | [17] |
|  | ApUR2 | TTTCTCATTCTTCAGATGATTTGG | 55.4 | 55 |  | [17] |

## RT-PCR analysis (HotStarTaq *Plus* DNA Polymerase, Qiagen 203605)

PCR mix (25 µl)

10x buffer (coloured) 2,5 µl

MgCl2 (25 mM) 1 µl final 1 mM

dNTPs (10 mM each) 0.5 µl final 0.2 mM

Forward primer (100 µM) 0.5 µl final 2 µM

Reversed primer (100 µM) 0.5 µl final 2 µM

*Taq* (5 U/µl) 0.2 µl final 1 U

ddH2O 18,8 µl

template (cDNA 100x) 1 µl

PCR program

Hot start 5 min @ 95 °C

Amplification (35x) 30 s @ 94 °C

30 s @ Ta (see primer look-up table above)

30s/45s/1min @ 72 °C

(0-500bp: 30 s, 501-750bp: 45 s, 751-1000bp: 1 min)

Extension 10 min @ 72 °C

HOLD 4°C

## Proof-reading (Platinum^TM^ *Pfx* DNA Polymerase, Life Technologies, 11708013)

PCR mix (25 µl)

10x buffer 2,5 µl

MgSO4 (50 mM) 0.5 µl final 1 mM

dNTPs (10 mM each) 0.5 µl final 0.2 mM

Forward primer (100 µM) 0.5 µl final 2 µM

Reversed primer (100 µM) 0.5 µl final 2 µM

*Pfx* (2.5 U/µl) 0.4 µl final 1 U

ddH2O 19,1 µl

template (cDNA 100x) 1 µl

PCR program

Hot start 5 min @ 95 °C

Amplification (35x) 30 s @ 94 °C

30 s @ Ta (see primer look-up table above)

30s/45s/1min @ 68 °C

(0-500bp: 30 s, 501-750bp: 45 s, 751-1000bp: 1 min)

Extension 10 min @ 68 °C

HOLD 4°C

*Before TOPO-TA cloning, add 1 µl Taq and incubate 10 min @ 72°C to ensure A-overhangs.*

## Difficult templates (Expand^TM^ Long Template PCR System, Roche, /)

now Sigma, 000000011681834001

PCR mix (50 µl)

10x buffer 2 5 µl

dNTPs (10 mM each) 1.75 µl final 350 µM

Forward primer (10 µM) 1.5 µl final 0.3 µM

Reversed primer (10 µM) 1.5 µl final 0.3 µM

Polymerase (5 U/µl) 0.75 µl final 3.75 U

ddH2O 38.5 µl

template (cDNA 100x) 1 µl

(gDNA 1x-10x)

PCR program

Hot start 2 min @ 95 °C

Amplification1 (10x) 10 s @ 94 °C

30 s @ Ta (see primer look-up table above)

4 min @ 68 °C

Amplification2 (25x) 10s @ 94 °C

30 s @ Ta (see primer look-up table above)

4 min 20 s @ 68 ° C (incrementing 20 s per cycle)

Extension 7 min @ 68 °C

HOLD 4°C

*Before TOPO-TA cloning, add 1 µl Taq and incubate 10 min @ 72°C to ensure A-overhangs.*

# References

1. Walsh, P.S., D.A. Metzger, and R. Higuchi, *Chelex 100 as a medium for simple extraction of DNA for PCR-based typing from forensic material.* Biotechniques, 1991. **10**(4): p. 506-13.

2. Stucky, B.J., *SeqTrace: a graphical tool for rapidly processing DNA sequencing chromatograms.* J Biomol Tech, 2012. **23**(3): p. 90-3.

3. Ratnasingham, S. and P.D.N. Hebert, *BOLD: The Barcode of Life Data System (*[*www.barcodinglife.org)*](http://www.barcodinglife.org))*.* Molecular Ecology Notes, 2007. **7**(3): p. 355-364.

4. Smith, M.A., et al., *Wolbachia and DNA barcoding insects: patterns, potential, and problems.* PLoS One, 2012. **7**(5): p. e36514.

5. Bioinformatics, B. *FastQC v0.11.3*. Accessed 2 Sept 2015]; Available from: <http://www.bioinformatics.bbsrc.ac.uk/projects/fastqc>.

6. Grabherr, M.G., et al., *Full-length transcriptome assembly from RNA-Seq data without a reference genome.* Nat Biotechnol, 2011. **29**(7): p. 644-52.

7. Camacho, C., et al., *BLAST+: architecture and applications.* BMC Bioinformatics, 2009. **10**: p. 421.

8. Tange, O., *GNU Parallel - The Command-Line Power Tool.* The USENIX Magazine, 2011. **February 2011**: p. 42-47.

9. Buchfink, B., C. Xie, and D.H. Huson, *Fast and sensitive protein alignment using DIAMOND.* Nature Methods, 2015. **12**(1): p. 59-60.

10. Huson, D.H., et al., *MEGAN analysis of metagenomic data.* Genome Research, 2007. **17**(3): p. 377-386.

11. Kim, D., B. Langmead, and S.L. Salzberg, *HISAT: a fast spliced aligner with low memory requirements.* Nat Methods, 2015. **12**(4): p. 357-60.

12. Team, R.D.C. *R: A Language and Environment for Statistical Computing*. R Foundation for Statistical Computing, Vienna, Austria 2011; Available from: <http://www.R-project.org/>.

13. Wickham, H., *ggplot2: Elegant Graphics for Data Analysis*. 2009, Springer-Verlag New York.

14. Gauthier, L., et al., *Viruses associated with ovarian degeneration in Apis mellifera L. queens.* PLoS One, 2011. **6**(1): p. e16217.

15. Singh, R., et al., *RNA Viruses in Hymenopteran Pollinators: Evidence of Inter-Taxa Virus Transmission via Pollen and Potential Impact on Non-Apis Hymenopteran Species.* Plos One, 2010. **5**(12).

16. Vossbrinck, C.R., et al., *Ribosomal DNA sequences of Encephalitozoon hellem and Encephalitozoon cuniculi: species identification and phylogenetic construction.* J Eukaryot Microbiol, 1993. **40**(3): p. 354-62.

17. Meeus, I., et al., *Multiplex PCR detection of slowly-evolving trypanosomatids and neogregarines in bumblebees using broad-range primers.* J Appl Microbiol, 2010. **109**(1): p. 107-15.
